# Phenylpiperazine 5,5-Dimethylhydantoin Derivatives as First Synthetic Inhibitors of Msr(A) Efflux Pump in *Staphylococcus epidermidis*

**DOI:** 10.3390/molecules25173788

**Published:** 2020-08-20

**Authors:** Karolina Witek, Gniewomir Latacz, Aneta Kaczor, Joanna Czekajewska, Ewa Żesławska, Anna Chudzik, Elżbieta Karczewska, Wojciech Nitek, Katarzyna Kieć-Kononowicz, Jadwiga Handzlik

**Affiliations:** 1 Department of Technology and Biotechnology of Drugs, Jagiellonian University, Medical College, ul. Medyczna 9, 30-688 Krakow, Poland; karolina.witek@uj.edu.pl (K.W.); glatacz@cm-uj.krakow.pl (G.L.); aneta.kaczor@doctoral.uj.edu.pl (A.K.); ankachudzik@yahoo.com (A.C.); mfkonono@cyf-kr.edu.pl (K.K.-K.); 2 Department of Pharmaceutical Microbiology, Jagiellonian University, Medical College, ul. Medyczna 9, 30-688 Krakow, Poland; j.czekajewska@uj.edu.pl (J.C.); elzbieta.karczewska@uj.edu.pl (E.K.); 3 Institute of Biology, Pedagogical University of Krakow, ul. Podchorążych 2, 30-084 Krakow, Poland; ewa.zeslawska@up.krakow.pl; 4 Faculty of Chemistry, Jagiellonian University, ul. Gronostajowa 2, 30-387 Krakow, Poland; nitek@chemia.uj.edu.pl

**Keywords:** *Staphylococcus epidermidis*, efflux pump, Msr(A), phenylpiperazine 5,5-dimethylhydantoins, efflux pump inhibitors, EPIs, X-ray crystallography

## Abstract

Herein, 15 phenylpiperazine 3-benzyl-5,5-dimethylhydantoin derivatives (**1**–**15**) were screened for modulatory activity towards Msr(A) efflux pump present in *S. epidermidis* bacteria. Synthesis, crystallographic analysis, biological studies in vitro and structure–activity relationship (SAR) analysis were performed. The efflux pump inhibitory (EPI) potency was determined by employing ethidium bromide accumulation assay in both Msr(A) efflux pump overexpressed (K/14/1345) and deficient (ATCC 12228) *S. epidermidis* strains. The series of compounds was also evaluated for the capacity to reduce the resistance of K/14/1345 strain to erythromycin, a known substrate of Msr(A). The study identified five strong modulators for Msr(A) in *S. epidermidis*. The 2,4-dichlorobenzyl-hydantoin derivative **9** was found as the most potent EPI, inhibiting the efflux activity in K/14/1345 at a concentration as low as 15.63 µM. Crystallography-supported SAR analysis indicated structural properties that may be responsible for the activity found. This study identified the first synthetic compounds able to inhibit Msr(A) efflux pump transporter in *S. epidermidis*. Thus, the hydantoin-derived molecules found can be an attractive group in search for antibiotic adjuvants acting via Msr(A) transporter.

## 1. Introduction

The Gram-positive bacteria *Staphylococcus epidermidis* belong to coagulase-negative staphylococci (CoNS) [1]. The bacteria are highly abundant on the skin and mucous membranes of humans and compete with other potentially harmful microorganisms, such as *S. aureus*, to maintain healthy skin microflora. For this reason, in the past, *S. epidermidis* was considered to be an important non-pathogenic commensal organism. However, it became evident that this natural skin colonizer is a true opportunistic pathogen, particularly in critically ill immunocompromised patients and those with indwelling foreign devices [2]. Indeed, *S. epidermidis* has been recognized as the leading causative agent of implanted medical device-related infections (e.g., intravascular catheter or prosthetic joint infections) in the hospital settings. Nosocomial infections caused by *S. epidermidis* have gained considerable attention in recent years due to an increase of resistance of the bacterium to antimicrobial drugs [2,3]. Currently, between 75% and 90% of *S. epidermidis* strains isolated in hospitals around the world are reported to be methicillin-resistant [1,2]. Acquisition of methicillin-resistance renders most β-lactam antibiotics, such as penicillins, cephalosporins (except for the fifth generation drugs), carbapenems and monobactams, useless in the therapy of staphylococcal infections [4]. In the presence of β-lactam resistance and in patients allergic to penicillin, the first-line alternative treatment is macrolide-lincosamide-streptogramin B (MLS_B_) group of antibiotics [5,6,7]. Although possessing different chemical structures, the mechanism of action of these antibacterials is similar. They act as protein synthesis inhibitors through interaction with the 23S rRNA component of the large 50S ribosomal subunit that is involved in the regulation of translation accuracy [8,9]. Since macrolides, lincosamides and streptogramins B share the same binding site, conformational changes in the ribosome lead to the development of cross-resistance of bacteria to these three groups of antibiotics [9]. The widespread use of MLS_B_ antibiotics over the last twenty years resulted in the number of staphylococcal strains acquiring resistance to these drugs [10]. The lack of susceptibility of staphylococci to MLS_B_ antibacterials is mostly attributed to the following mechanisms: (i) target site modification; (ii) active efflux; and (iii) enzymatic inactivation. Depending on the occurrence of the aforementioned mechanisms, different resistance phenotypes are expressed. The most prevalent ones are MLS_B,_ which, in *S. aureus* and CoNS, arise from the methylation of adenine within 23S rRNA ribosomal subunits mediated by the erythromycin resistance methylase genes *ermA* and *ermC* [11]. The expression of MLS_B_ phenotype can be either constitutive (cMLS_B_) and confer a high level of cross-resistance to all MLS_B_ antibiotics or inducible (iMLS_B_) occurring when the production of methylases is a result of the induction of *erm* genes that consequently leads to the lack of susceptibility of bacteria to macrolides with a 14- and 15-membered lactone ring (e.g., erythromycin, azithromycin or clarithromycin) [12]. The active efflux of antibiotics is determined by *msr* genes (predominantly *msrA*) and is associated with the MS_B_ phenotype. It confers resistance only to 14- and 15-membered ring macrolides and streptogramins B [13]. Since 16-membered ring macrolides (josamycin and spiramycin) and lincosamides are neither inducers nor substrates for the pump, these antibiotics remain active. The *msrA* resistance determinant was reported for the first time in *S. epidermidis*, and, since then, it has also been found in *S. aureus* and other staphylococcal species [14]. The gene encodes Msr(A) efflux pump that is a member of the ATP-binding-cassette (ABC) transporter superfamily comprising important mediators of drug resistance in microorganisms [13]. Notably, the study conducted by Eady et al. indicated that the presence of *msr*(*A*) was associated with erythromycin resistance in 36.4% clinical CoNS [15]. Given the clinical importance of efflux pumps in bacterial drug resistance, attempts have been made to identify potent EPIs of Msr(A) transporter in staphylococci. By means of fluorometric assays, it has been demonstrated that carnosic acid, an abietane diterpene presented in the popular herbs rosemary (*Rosmarinus officinalis*) and sage (*Salvia officinalis*), is able to modulate Msr(A) transporter in Msr(A)-overexpressing *S. aureus* strain RN4220. Furthermore, it has been shown that this natural compound is a good potentiator of erythromycin activity in the resistant to this antibiotic *S. aureus* RN4220 [16]. However, to the best of our knowledge, no synthetic compound that displays EPI effect on Msr(A) exporter in *S. epidermidis* has been described so far.

During the course of efforts to discover novel efflux pump inhibitors with antibiotic adjuvant activity, our research group has found that some phenylpiperazine 5,5-dimethylhydantoin derivatives act as inhibitors of intrinsic and overexpressed efflux transporters present in *S. aureus* strains [17]. Based on that interesting outcome, seven phenylpiperazine 5,5-dimethylhydantoins, which were previously tested for their EPI activity in *S. aureus* pathogen (**1**–**7**), were selected for this approach (Table 1). Thus, the compounds (**1**–**7**) and their new derivatives synthesized within this study (**8**–**15**, Table 1) were evaluated for the ability to block the Msr(A) efflux pump in *S. epidermidis* K/14/1345 clinical isolate harboring MS_B_ resistance phenotype. 

Moreover, the effect displayed by the series of derivatives in Msr(A)-overexpressing *S. epidermidis* K/14/1345 was compared to that assessed in Msr(A)-deficient *S. epidermidis* strain ATCC 12228. The EPI activity of the 5,5-dimethylhydantoins was detected by the aid of the real-time ethidium bromide (EtBr) accumulation method. Antibiotic adjuvant properties of the compounds have been verified in the MIC reduction assay, in which their capacity to reduce or reverse the resistance of *S. epidermidis* K/14/1345 to erythromycin, a well-known substrate of Msr(A) transporter, was assessed. For representative compounds, crystallographic analyses were performed to enrich SAR discussion. Thus, the study was focused on synthesis, X-ray crystallography, comprehensive biological assays and SAR analysis for the series of phenylpiperazine hydantoins **1**–**15** given in Table 1.

## 2. Results

### 2.1. Chemistry 

**Scheme 1:**
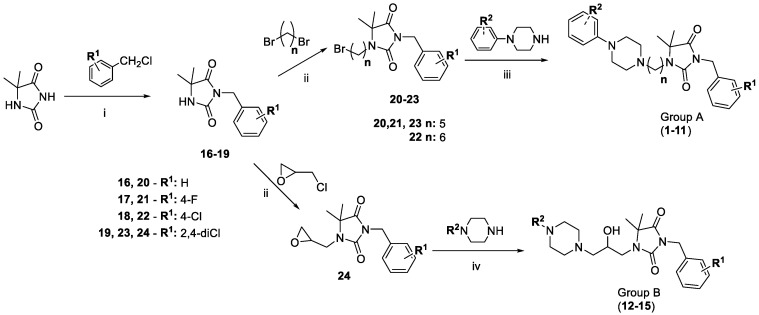
Synthesis route for compounds **1**–**15**: (**i**) K_2_CO_3_, TEBA, acetone, reflux, 4 h; (**ii**) K_2_CO_3_, TEBA, acetone, rt, 120–144 h; (**iii**) K_2_CO_3_, acetone, reflux, 4–11 h; and (**iv**) mw, 300–450 W, 3–7 min.

Synthesis of compounds **1**–**15** was performed according to Scheme 1. Details of synthesis for compounds **1**–**7** and intermediates **16**, **17**, **19**–**21**, **23** were described elsewhere [18]. The synthesis route consists of three steps: (i) N3-alkylation; (ii) N1-alkylation; and (iii or iv) N-alkylation using 1-phenylpiperazine derivatives. Firstly, N3-alkylation (i) between 5,5-dimethylhydantoin and benzyl chloride derivatives was performed. In this reaction, 3-benzyl-5,5-dimethylimidazolidine-2,4-diones (**16**–**19**) were obtained. The intermediates (**16**–**19**) were used for N1-alkylation (ii), which was performed with dibromo-alkanes (**20**–**23**, Group A) or 2-(chloromethyl)oxirane (**24**, Group B), respectively. This reaction provided intermediates **20**–**24**. In the last step (iii or iv), N-alkylations of 1-phenylpiperazine derivatives were performed, using either a suitable bromoalkyl intermediate (**20**–**23**) or the oxirane derivative **24**. In the first case, the process proceeding in basic conditions gave products **1**–**11** (Group A), while the reaction with **24** occurred via oxirane ring opening under microwave irradiation in solvent-free conditions to give **12**–**15** (Group B, Scheme 1). 

### 2.2. X-ray Crystallographic Studies

**Figure 1:**
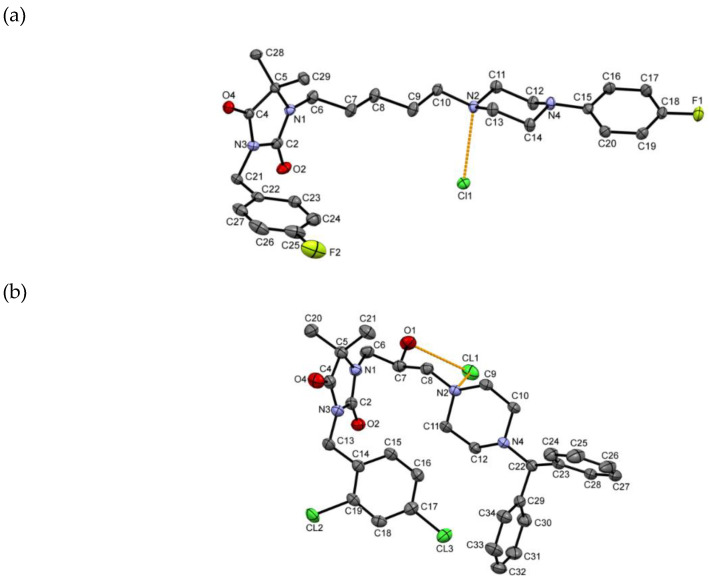
The molecular geometry with the atom numbering schemes of: (**a**) **6**; and (**b**) **15**. Hydrogen atoms have been omitted for clarity. Displacement ellipsoids are drawn at the 50% probability level. Orange dashed lines show hydrogen bonds between the chloride anion and protonated N2 atom for both structures and between the chloride anion and hydroxy group in **15**.

For two representative compounds, displaying EPI action (**6**) and inactive one (**15**), X-ray crystallographic analysis was carried out. The molecular geometry in the crystal structures of **6** and **15** together with the atom numbering schemes is shown in Figure 1. 

Both molecules contain 3-benzyl-5,5-dimethylhydantoin moiety with different substituents in the aromatic ring (4-F for **6** and 2,4-di-Cl for **15**). The angle between the planes of aromatic and hydantoin rings is 80.52(5)° for **6** and 71.56(7)° for **15**. The introduction of the second substituent in position 2 reduced the value of this angle. None of these halogen atoms is involved in intermolecular halogen bonds. The investigated compounds differ in the linker between hydantoin and piperazine rings. The linker of **6** consists of five methylene units and adopts an extended conformation, while the linker of **15** has only three methylene units with hydroxy group located at the second carbon atom. In this molecule, the linker adopts bent conformation. 

In the presented crystal structures, the piperazine ring adopts chair conformation with an equatorial position of substituents at N2 and N4 atoms. The angle between the planes of aromatic and the carbon atoms of piperazine rings in **6** is 26.22(8)°. Similar values of this angle have been also observed in other crystal structures containing 4-(4-fluorobenzene)piperazine moiety deposited in Cambridge Structure Database [19]. In **15**, diphenylmethyl substituent at piperazine ring adopts conformation, which is similar to other crystal structures containing this moiety [19].

In both structures, the chlorine anion is involved in charge assisted hydrogen bond with protonated N2 atom (Figure 1). In the crystal of **15**, the chlorine ion is also engaged in interaction with oxygen atom of hydroxy group. The intermolecular interactions in the crystals are dominated by N-H···Cl, C-H···Cl and C-H···O contacts. Additionally, C-H···N and C-H···F in **6** and O-H···Cl in **15** contacts are observed. The parameters of these interactions are listed in Table 2.

### 2.3. Biological Assays

#### 2.3.1. Direct Antibacterial Activity 

In the first step of the biological assays, direct antibacterial activity of 15 phenylpiperazine 5,5-dimethylhydantoin derivatives (**1**–**15**) was determined against *S. epidermidis* K/14/1345 and *S. epidermidis* ATCC 12228 strains to select the concentrations of the compounds at which their antibiotic adjuvant effect should be tested. The macrolide antibiotic, erythromycin, was used as reference in the assay. For most of the compounds (**1**, **2**, **4**–**6** and **8**–**15**), precipitation in MH II broth was observed at the highest concentrations tested. For these molecules, MICs could not be established precisely but was approximated as a higher value than the highest possible concentration at which the growth of bacteria was still observed. MIC values of the series of phenylpiperazine 5,5-dimethylhydantoin derivatives are shown in Table 3. The results of the study indicate that phenylpiperazine 5,5-dimethylhydantoin derivatives tested exerted very weak (**1**–**8** and **12**–**14**) to moderate (**9**–**11** and **15**) antibacterial activity against *S. epidermidis* strains selected for the study. Among the 15 derivatives, the most remarkable antibacterial effect was found for compound **15**, which affected bacterial growth at the concentration <10 µM (<5 µg/mL) in the case of reference strain (Table 3). The significant difference in MIC values of erythromycin against *S. epidermidis* strains K/14/1345 and ATCC 12228 was in high accordance with the resistant and susceptible phenotypes of these strains, respectively.

#### 2.3.2. Inhibitory Action on Msr(A) Efflux Pump 

To establish whether compounds tested have the EPI activity on the Msr(A) efflux transporter, accumulation assay, which employed ethidium bromide (EtBr), the dye-substrate of the pump, was performed. For this task, EtBr at a final concentration of 1 μg/mL and compounds tested at a final concentration corresponding to 1/4 or 1/2 of their MIC values (the highest possible concentration that did not inhibit bacterial growth in the case of precipitating compounds) were added to the bacterial suspensions. The experiment was performed under conditions that allow maximum efflux (the presence of glucose, which is a source of energy for the pump, and the temperature of 37 °C).

**Figure 2:**
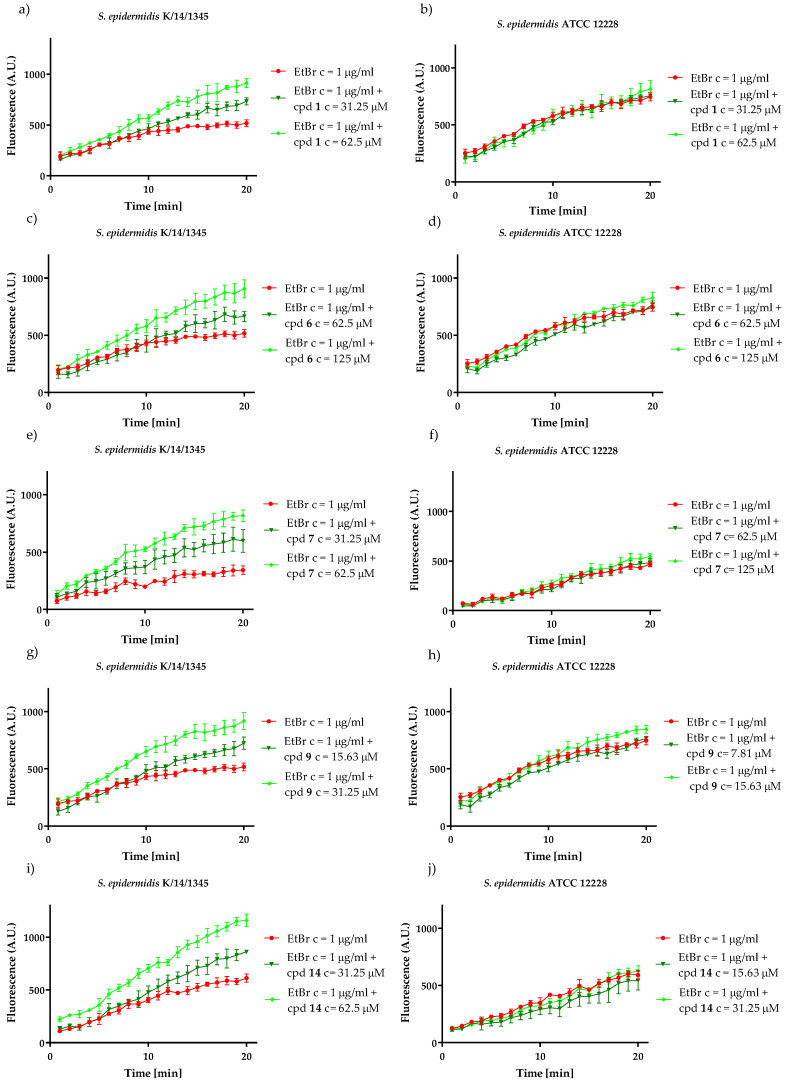
Influence of compounds **1** (**a**,**b**), **6** (**c**,**d**), **7** (**e**,**f**), **9** (**g**,**h**) and **14** (**i**,**j**) tested at the concentrations corresponding to 1/4 and 1/2 of their MIC values on the accumulation of EtBr (1 µg/mL) in Msr(A)-overexpressing *S. epidermidis* strain K/14/1345 and Msr(A)-deficient *S. epidermidis* strain ATCC 12228. Each data point expresses the mean ± standard deviation (SD) from four replicates.

**Figure 3:**
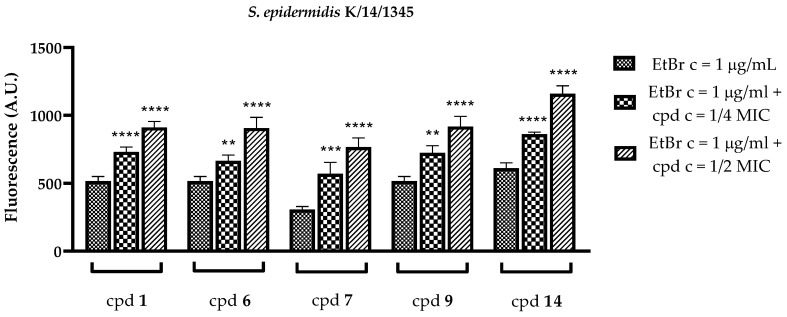
Fluorescence intensity of EtBr (1 µg/mL) after addition of compounds **1**, **6**, **7**, **9** and **14** at 20-min time point in *S. epidermidis* K/14/1345. Statistical significance was calculated using a one-way analysis of ANOVA, followed by the multiple comparison test (** *p* < 0.05, *** *p* < 0.001, **** *p* < 0.0001).

The data obtained in accumulation assay enabled identifying five strong EPIs (**1**, **6**, **7**, **9** and **14**) of Msr(A) efflux transporter. The level of accumulation of EtBr in the absence and presence of active compounds in Msr(A)-overexpressing *S. epidermidis* strain K/14/1345 and Msr(A)-deficient *S. epidermidis* strain ATCC 12228 is shown in Figure 2 and Figure 3.

As shown in Figure 2 and Figure 3, the addition of compounds **1**, **6**, **7**, **9** and **14** caused a statistically significant rise of the fluorescence intensity in Msr(A)-overexpressing *S. epidermidis* strain K/14/1345. The increase in the intracellular concentrations of the dye in combination with the aforementioned compounds was dose-dependent with the most prominent effect observed at the concentration of 1/2 of their MIC values. To compare the modulatory effect of new hydantoin-derived EPIs towards Msr(A) protein, relative fluorescence index (RFI) was calculated using the relative final fluorescence (RFF) at 20-min time point of the EtBr accumulation curve. The RFF parameter is an indicator of the capacity of a compound to modulate the efflux pump activity of bacterium. The greater is the RFF, the greater is the degree of inhibition of an efflux pump transporter and, therefore, the more potent is the EPI activity of a given compound tested [17]. 

Since EPI potency of 5,5-dimethylhydantoin derivatives depended on the concentration of compounds used in the study, specific activity (SA) of the inhibition of an efflux pump was determined. The parameter was calculated by dividing the RFI by the number of micromoles of a molecule evaluated in the accumulation assay [17]. The results of the analysis performed on the EPI effect of the hydantoin derivatives are given in Table 4. As in the case of RFF and RFI, the higher is the SA of a compound tested, the greater is the inhibitory effect on a given efflux pump transporter. 

The data obtained show that, among all identified EPIs of Msr(A) efflux transporter, the most prominent effect was observed for the member of Group A, compound **9**. The compound increased significantly the accumulation of EtBr in *S. epidermidis* K/14/1345 overproducing Msr(A) efflux pump at the lowest concentrations (15.63, 25 and 31.25 µM) tested (SA = 24.82). The effectiveness of **9** was followed by the one of compound **7** (SA = 22.16). After 20 min of incubation, compound **7**, tested at the concentration of 62.5 µM, increased more than two-fold the EtBr accumulation in *S. epidermidis* strain K/14/1345. The influence of the compound on the EtBr accumulation at the concentration of 31.25 µM was less pronounced, but also significant. Compound **14** was the most active EPI of Msr(A) found in Group B. The compound showed high Msr(A) inhibitory efficacy at the concentration of both 62.5 and 31.25 µM. The strength of the inhibitory effect of **7**, **9** and **14** was followed by the activity of two members of Group A: compounds **1** and **6**.

Remarkably, compounds **1**, **6**, **7**, **9** and **14** did not influence EtBr accumulation in Msr(A)-deficient *S. epidermidis* strain ATCC 12228. Remaining 5,5-dimethylhydantoin derivatives, including **2**–**5**, **8**, **10**–**13** and **15**, were devoid of inhibitory activity on the extrusion of the dye from both Msr(A)-overexpressing and Msr(A)-deficient strains (Figures S1–S20). At the same time, the addition of compound **15** to EtBr decreased the fluorescence of the dye when tested in both *S. epidermidis* K/14/1345 and *S. epidermidis* ATCC 12228. The effect might be due to molecular interactions between fluorophore and **15** that resulted in increased quenching of fluorescence. Importantly, additional controls performed indicated that none of compounds tested shown its own fluorescence and, thus, did not interfere with the EtBr fluorescence measured in the accumulation assay.

It should be noted that the increase in fluorescence intensity of EtBr in the absence and presence of compounds tested in *S. epidermidis* K/14/1345 isolate was relatively slower than that noted for the ATCC strain (Figure 2 and Figures S1–S20). This observation suggests that the rate of EtBr accumulation was less pronounced in the strain that overexpresses the Msr(A) efflux pump and agrees with the general premise that the higher is the level of efflux, the lower is the accumulation of a substrate of an efflux pump inside the bacterial cell [20].

#### 2.3.3. Influence on the Activity of Erythromycin 

The ability of the series of phenylpiperazine 5,5-dimethylhydantoin derivatives (**1**–**15**) to enhance the antibacterial activity of erythromycin against both *S. epidermidis* K/14/1345 and *S. epidermidis* ATCC 12228 was investigated. The compounds were tested at the sublethal concentrations of 1/4 their respective MICs. In each experiment, the control of the efficacy of erythromycin against *S. epidermidis* strains in the absence of compounds tested was also performed. Results obtained in the assay show that none of the phenylpiperazine 5,5-dimethylhydantoins could strengthen the effect of Msr(A) substrate, erythromycin, against resistant to this antibiotic *S. epidermidis* K/14/1345 clinical isolate. Similarly, the compounds did not influence the activity of erythromycin in susceptible *S. epidermidis* ATCC 12228 (Table S1). 

## 3. Discussion

In the present work, the influence of 15 phenylpiperazine 5,5-dimethylhydantoin derivatives on Msr(A) efflux pump transporter in *S. epidermidis* strain K/14/1345 possessing MS_B_ phenotype was evaluated. The capacity of compounds to reduce the extrusion of EtBr through the inhibition of the pump was analyzed by the use of the real-time accumulation assay in which 5,5-dimethylhydantoin derivatives were tested at the doses nontoxic for the bacteria. The results of the assay show that five derivatives (**1**, **6**, **7**, **9** and **14**) significantly increase the accumulation of EtBr in Msr(A)-overexpressing *S. epidermidis* isolate. The most potent EPI activity was noted for compound **9**, containing fluorine atom at positions *ortho* of the phenylpiperazine moiety connected with hydantoin core by pentyl linker and 2,4-dichlorobenzyl moiety at hydantoin position 3. This molecule at the concentrations as low as 15.63 and 31.25 µM highly effectively inhibited the extrusion of the fluorescent dye from *S. epidermidis* K/14/1345 overproducing Msr(A) protein. A similar modulatory effect, but at a higher concentration tested, was observed for derivative **7**, which possesses two fluorine atoms at positions 2 and 4 of the phenylpiperazine moiety linked to 5,5-dimethylhydantoin skeleton by pentyl chain. Compound **7** at the concentration of 62.5 µM reduced efflux activity of Msr(A) more than two-fold in *S. epidermidis* K/14/1345 strain. Potent EPI activity of **7** was already confirmed by the previous study of Amaral et al. where the compound displayed high effectiveness in inhibiting overproduced Qac transporter present in *S. aureus* strain HPV-107 and constitutively expressed efflux system of the wild-type *S. aureus* strain ATCC 25923 [17]. The fact that **7** has the capacity to interact with different types of efflux pumps expressed in staphylococci indicates that the compound possesses a multitarget specificity for these membrane-related transporters. The EPI property towards Msr(A) exporter amongst remaining phenylpiperazine 5,5-dimethylhydantoin derivatives was shown to decrease as follows: **14** > **1** > **6**. Remarkably, it has been demonstrated that the identified inhibitors of Msr(A) transporter protein did not influence the accumulation of EtBr in *S. epidermidis* strain ATCC 12228. 

It is intriguing that the found EPIs structurally differ from each other more significantly than from some inactive members of the series (**1**–**15**). Considering the three modified structural moieties (Table 1), some structure–activity effects may be noted. For the influence of the phenylpiperazine moiety substituents, the following EPI-activity order can be seen: 2-F > 2,4-diCl > 2,4-diF > 4-F. On the other hand, 2,4-diCl-, 4-F- and lack of substituents were beneficial in the case of benzyl moiety at the hydantoin position 3. Most of the active EPIs contain 5-carbon linker (**1**, **6**, **7** and **9**). Although one exception, i.e., the hydoxypropyl derivative **14**, is placed in the exact middle of this range, other compounds with the hydroxypropyl linker were totally inactive. This can suggest a beneficial role of the C5-linker, predominant for EPI properties in comparison to either the shorter branched hydroxypropyl linker (**12**–**15**) or the longer one (C6, compound **8**). 

Despite the relevant EPI activity of five phenylpiperazine 5,5-dimethylhydantoin derivatives in the EtBr accumulation assay, the compounds tested were not able to increase the effectiveness of erythromycin against resistant to this drug *S. epidermidis* strain K/14/1345. The reason for discrepancies in the behavior of the compounds in the MIC reduction assay and the EtBr accumulation assay might be explained by the significant differences in the experimental conditions used in the MIC reduction assay and in the real-time accumulation assay. The latter is carried out in a minimal volume of medium and over a short period of time, while the test that is based on the determination of the capacity of molecules to increase the susceptibility of a strain to a given antibiotic is performed in a complete medium and over 18 h of incubation. Bearing in mind that during the MIC reduction assay bacteria are exposed to an antibiotic and a compound for an extended period of time, the test provides favorable conditions for the inducement of genes responsible for efflux pumps overexpression. Ultimately, an antibiotic or a compound selected for the experiment may promote the production of efflux pump units leading to the reduction of the efficacy of a potential EPI under investigation [17,21]. 

The analysis of the obtained results does not give an evident SAR. In general, two aromatic ends with hydrophobic halogen substituents seem to favor the EPI properties for Msr(A). This remains the structural traits required for modulators of Pgp that also represents ABC transporter family. Within the considered series (**1**–**15**), there is a “black swan” **15** that does not possess phenylpiperazine, but the benzhydrylpiperazine moiety. This compound stood out from the rest with its significant antibacterial activity and may constitute a lead structure for a new separate research direction, which can be supported by 3D-structure details coming from the X-ray analysis performed within the present study.

## 4. Experimental

### 4.1. Chemistry

Reagents were purchased from Alfa Aesar (Karlsruhe, Germany) or Sigma Aldrich (Darmstadt, Germany). Reaction progress was verified using thin layer chromatography (TLC), which was carried out on 0.2 mm Merck silica gel 60 F254 plates. Spots were visualized by UV light. Melting points (m.p.) were determined using MEL-TEMP II apparatus (LD Inc., Long Beach, CA, USA) and are uncorrected. The ^1^H-NMR and ^13^C-NMR spectra were obtained on a Mercury-VX 300 Mz spectrometer (Varian, Palo Alto, CA, USA) in DMSO-d_6_. Chemical shifts in ^1^H-NMR spectra were reported in parts per million (ppm) on the δ scale using the solvent signal as an internal standard. Data are reported as follows: Chemical shift, multiplicity (s, singlet; br.s., broad singlet; d, doublet; d def., doublet deformated; t, triplet; t def., triplet deformated; q def., quarted deformated qui, quintet; m, multiplet), coupling constant J in Hertz (Hz), number of protons, proton’s position (Pp-Ph, phenyl connected to piperazine; Ph, phenyl of benzyl fragments; Pp, piperazine). Mass spectra were recorded on a UPLC-MS/MS system consisted of a Waters ACQUITY^®^UPLC^®^ (Waters Corporation, Milford, MA, USA) coupled to a Waters TQD mass spectrometer (electrospray ionization mode ESI-tandem quadrupole). Chromatographic separations were carried out using the Acquity UPLC BEH (bridged ethyl hybrid) C18 column, 2.1 × 100 mm, and 1.7 µm particle size, equipped with Acquity UPLC BEH C18 VanGuard precolumn (Waters Corporation, Milford, MA, USA), 2.1 × 5 mm, and 1.7 µm particle size. The column was maintained at 40 °C and eluted under gradient conditions from 95% to 0% of eluent A over 10 min, at a flow rate of 0.3 mL·min^−1^. Eluent A: water/formic acid (0.1%, *v*/*v*); eluent B: acetonitrile/formic acid (0.1%, *v*/*v*). Chromatograms were made using Waters eλ PDA detector. Spectra were analyzed in the 200–700 nm range with 1.2 nm resolution and sampling rate 20 points/s. MS detection settings of Waters TQD mass spectrometer were as follows: source temperature 150 °C, desolvation temperature 350 °C desolvation gas flow rate 600 L·h^−1^, cone gas flow 100 L·h^−1^, capillary potential 3.00 kV and cone potential 40 V. Nitrogen was used for both nebulizing and drying gas. The data were obtained in a scan mode ranging from 50 to 1000 m/z in 0.5-s intervals. Data acquisition software was MassLynx V 4.1 (Waters Corporation, Milford, MA, USA). The UPLC/MS purity of all the final compounds was confirmed to be 95% or higher. Retention times (t_R_) are given in minutes. The UPLC/MS purity of all final compounds was determined (%). IR spectra (for compounds **9**–**11** to confirm their hydrochloride forms at the absence of NH^+^ proton peak in ^1^H-NMR) were recorded on a Jasco FT/IR-410 apparatus using KBr pellets and are reported in cm^−1^. Syntheses under microwave irradiation were performed in household microwave oven Samsung MW71B. Synthesis of compounds **1**–**7**, **16**–**17**, **19**–**21** and **23** was described earlier [18]. 

#### 4.1.1. 3-(4-Chlorobenzyl)-5,5-dimethylimidazolidine-2,4-dione (**18**)

5,5-Dimethylhydantoin (120 mmol, 15.37 g), potassium carbonate (34.73 mmol, 48.0 g), TEBA (15.8 mmol, 3.6 g) and acetone (480 mL) were heated in boiling point for 20 min. Then, 1-chloro-4-(chloromethyl)benzene (120 mmol, 19.32 g) dissolved in acetone (80 mL) was added. Mixture was heated in boiling point for 4 h and then mixing for 20 h. Filtration was performed and distilled water was added to filtrate. Product appeared as solid and another filtration was performed. White solid. Yield 67.0%; mp 108–110 °C. C_12_H_13_ClN_2_O_2_ MW 252.07. LC/MS±: (ESI) m/z [M+H] 253.25. ^1^H-NMR δ (ppm): 1.28 (s, 6H, 2 × 5-CH_3_), 4.50 (s, 2H, N3-CH_2_), 7.22–7.25 (d def., 2H, Ph-2,6-H), 7.37–7.40 (d def., 2H, Ph-3,5-H), 8.38 (s, 1H, N1-H).

#### 4.1.2. 3-(4-Chlorobenzyl)-1-(6-bromohexyll)-5,5-dimethylimidazolidine-2,4-dione (**22**)

3-(4-Chlorobenzyl)-5,5-dimethylimidazolidine-2,4-dione (**18**) (40 mmol, 10.1 g), potassium carbonate (115.77 mmol, 16.0 g), TEBA (5.27 mmol, 1.2 g) and acetone (80 mL) was heated for 30 min. Then, 1,6-dibromohexane (60 mmol, 14.63 g) dissolved in acetone (40 mL) was added. It was mixed for 144 h. Filtration was performed. Filtrate was evaporated, dichloromethane was added and mixture was extracted three times with 1% sodium hydroxide and twice with distilled water. Organic fraction was dried under sodium sulphate anhydrous, then filtrated and evaporated. To obtain pure product, column chromatography was performed in dichloromethane:acetone/10:1. Colourless oil. Yield 25.0%. C_18_H_24_BrClN_2_O_2_ MW 414.07. LC/MS±: (ESI) m/z [M + H] 415.03. ^1^H-NMR δ (ppm): 1.22–1.25 (t def., 1H, N3-CH_2_-CH_2_-CH_2_-CH_2a_), 1.28 (t def., 1H, N3-CH_2_-CH_2_-CH_2_-CH_2b_), 1.31 (s, 6H, 2 × 5-CH_3_), 1.37–1.41 (m, 2H, N3-CH_2_-CH_2_-CH_2_), 1.49–1.59 (m, 2H, N3-CH_2_-CH_2_), 1.73–1.82 (m, 2H, N3-CH_2_-CH_2_-CH_2_-CH_2_-CH_2_), 3.19–3.24 (t, J = 7.6 Hz, 2H, N1-CH_2_), 3.48–3.53 (t def., 2H, N3-CH_2_-CH_2_-CH_2_-CH_2_-CH_2_-CH_2_), 4.52 (s, 2H, N3-CH_2_), 7.22–7.24 (d def., 2H, Ph-2,6-H), 7.37–7.40 (d def., 2H, Ph-3,5-H). 

#### 4.1.3. 3-(2,4-Dichlorobenzyl)-5,5-dimethyl-1-(oxiran-2-ylmethyl)imidazolidine-2,4-dione (**24**)

3-(2,4-Dichlorobenzyl)-5,5-dimethylimidazolidine-2,4-dione (**19**) (100 mmol, 28.71 g), potassium carbonate (289.4 mmol, 40.0 g), TEBA (13.17 mmol, 3.0 g) and acetone (200 mL) were heated for 30 min. Then, 2-(chloromethyl)oxirane (110 mmol, 10.18 g) dissolved and acetone (96 mL) was added. Mixture was mixing in room temperature for 120 h. Filtration was performed. Filtrate was evaporated. To obtain pure product, column chromatography was performed in dichloromethane:acetone/10:1. Colourless oil. Yield 39.0%. C_15_H_16_Cl_2_N_2_O_3_ MW 342.05. LC/MS±: purity 93.35% t_R_ = 6.60, (ESI) m/z [M + H] 344.21. ^1^H-NMR δ (ppm): 1.34–1.40 (s, 6H, 2 × 5-CH_3_), 2.61–2.64 (m, 1H, N1-CH_2_-CH-CH_2_), 2.75–2.78 (m, 1H, N1-CH_2_-CH-CH_2_), 3.04–3.09 (m, 1H, N1-CH_2_-CH), 3.17–3.24 (m, 1H, N1-CH_2a_), 3.61–3.68 (m, 1H, N1-CH_2b_), 4.62 (s, 2H, N3-CH_2_), 7.17–7.20 (d def., 1H, Ph-6-H), 7.38–7.42 (d def., 1H, Benz-5-H), 7.62–7.63 (d def., 1H, Ph-3-H).

#### 4.1.4. General Procedure to Obtain Final Products (**8**–**11**)

1-Phenylpiperazine derivatives (3–4 mmol), potassium carbonate (10–13 mmol, 1.35–1.80 g), acetone (11–15 mL) and 3-((di)chlorobenzyl)-1-bromoalkyl-5,5-dimethylimidazolidine-2,4-dione derivatives (**22**–**23**) (4.8–5.8 mmol) dissolved in acetone (10–12.5 mL) were heated in boiling point for 4–11 h. Filtration of inorganic compound was performed. Filtrate was evaporated, dichloromethane was added and mixture was extracted 2–3 times with 1% hydrochloric acid (20 mL). Organic fraction was dried under sodium sulphate anhydrous, then filtrated and evaporated. For compounds **9**–**11**, crystallization using methanol was performed. Solid products were obtained with Method A or B. Method A: The pure product precipitated after saturation with gaseous hydrochloric acid. When Method A did not provide a precipitate of an expected hydrochloric salt, Method B was further involved, according to the following procedure: the solution was evaporated, a residue dissolved in anhydrous ethanol. The cold diethyl ether (0 °C) was added until a precipitation appeared, followed by mixing in a cork flask for 30 min. The mixture was cooled at <−10 °C for 48–168 h, filtered off and the precipitate washed with 2–5 mL of cold ether or acetone to give the pure product of a desirable arylpiperazine hydantoin derivative.

##### 3-(4-Chlorobenzyl)-1-(6-(4-(2-fluorophenyl)piperazin-1-yl)hexyl)-5,5-dimethylimidazolidine-2,4-dione hydrochloride (**8**)

1-(2-Fluorophenyl)piperazine (3 mmol, 0.54 g) and the 6-bromohexyl derivative **22** (5.8 mmol, 2.43 g) were used. Product was obtained with Method B. White solid. Yield 5.5%; mp 172–174 °C. C_28_H_37_Cl_2_FN_4_O_2_ MW 550.23. LC/MS±: purity 95.44% t_R_ = 5.92, (ESI) m/z [M + H] 515.56. ^1^H-NMR δ (ppm): 1.30–1.35 (m, 4H,N1-CH_2_-CH_2_-CH_2_-CH_2_), 1.32 (s, 6H, 2 × 5-CH_3,_), 1.50–1.65 (d def., 2H, N1-CH_2_--CH_2_-CH_2_-CH_2_-CH_2_), 1.72 (s, 2H, N1-CH_2_-CH_2_), 3.03–3.25 (m, 8H, Pp-CH_2_, Pp-2,6-H, N1-CH_2_), 3.43–3.53 (m, 4H, Pp-3,5-H), 4.53 (s, 2H, N3-CH_2_), 7.01–7.17 (m, 4H, Pp-Ph-3,4,5,6-H), 7.22–7.24 (d def., 2H, Ph-2,6-H), 7.37–7.40 (d def., 2H, Ph-3,5-H), 10.91 (br. s, 1H, NH^+^).

##### 3-(2,4-Dichlorobenzyl)-1-(5-(4-(2-fluorophenyl)piperazin-1-yl)pentyl)-5,5-dimethylimidazolidine-2,4-dione hydrochloride (**9**)

1-(2-Fluorophenyl)piperazine (4 mmol, 0.72 g) and the 5-bromopentyl derivative **23** (4.8 mmol, 2.00 g) were used. Product was obtained with Method B. White solid. Yield 6.0%; mp 172–174 °C. C_27_H_34_Cl_3_FN_4_O_2_ MW 570.17. LC/MS±: purity 98.68% t_R_ = 6.09, (ESI) m/z [M + H] 535.14. ^1^H-NMR δ (ppm): 1.22 (s, 2H, Pp-CH_2_-CH_2_-CH_2_), 1.37 (s, 6H, 2 × 5-CH_3_), 1.60–1.73 (m, 4H, Pp-CH_2_-CH_2_, N1-CH_2_-CH_2_), 3.06–3.14 (m, 6H, Pp-CH_2_, Pp-2,6-H), 3.24–3.29 (m, 2H, N1-CH_2_), 3.43–3.58 (m, 4H, Pp-3,5-H), 4.60 (s, 2H, N3-CH_2_), 7.02–7.17 (m, 5H, Pp-Ph-3,4,6-H, Ph-5,6-H), 7.39–7.43 (m, 1H, Pp-Ph-5-H), 7.63–7.64 (d def., 1H, Ph-3-H). IR (KBr) (cm^−1^): 3449 (NH), 3047 (CH Ar), 2933 (CH), 2358 (NH^+^), 1764.24 (C2=O), 1704 (C4=O), 1448 (C=C Ar).

##### 3-(2,4-Dichlorobenzyl)-1-(5-(4-(4-fluorophenyl)piperazin-1-yl)pentyl)-5,5-dimethylimidazolidine-2,4-dione hydrochloride (**10**)

1-(4-Fluorophenyl)piperazine (4 mmol, 0.72 g) and the 5-bromopentyl derivative **23** (4.8 mmol, 2.00 g) were used. Product was obtained with Method A. White solid. Yield 42.0%; mp 187–189 °C. C_27_H_34_Cl_3_FN_4_O_2_ MW 570.17. LC/MS±: purity 96.22% t_R_ = 6.06, (ESI) m/z [M + H] 535.14. ^1^H-NMR δ (ppm): 1.22 (s, 2H, Pp-CH_2_-CH_2_-CH_2_), 1.36 (s, 6H, 2 × 5-CH_3_), 1.60–1.73 (m, 4H, Pp-CH_2_-CH_2_, N1-CH_2_-CH_2_), 3.02–3.10 (m, 6H, Pp-CH_2_, Pp-2,6-H), 3.24–3.29 (m, 2H, N1-CH_2_), 3.51–3.72 (dd def., 4H, Pp-3,5-H), 4.60 (s, 2H, N3-CH_2_), 6.98–7.11 (m, 4H, Pp-Ph-2,3,5,6-H), 7.15–7.19 (d def., 1H, Ph-5-H), 7.39–7.43 (m, 1H, Ph-5-H), 7.63–7.64 (d def., 1H, Ph-3-H). IR (KBr) (cm^−1^): 3464 (NH), 3057 (CH Ar), 2982 (CH), 2444 (NH^+^), 1768 (C2=O), 1706 (C4=O), 1515 (C=C Ar).

##### 3-(2,4-Dichlorobenzyl)-1-(5-(4-(2,4-difluorophenyl)piperazin-1-yl)pentyl)-5,5-dimethylimidazolidine-2,4-dione hydrochloride (**11**)

1-(2,4-Difluorophenyl)piperazine (4 mmol, 0.79 g) and the 5-bromopentyl derivative **23** (5 mmol, 2.18 g) were used. Product was obtained with Method A. White solid. Yield 41.0%; mp 158–160 °C. C_27_H_33_Cl_3_F_2_N_4_O_2_ MW 588.16. LC/MS±: purity 98.38% t_R_ = 6.08, (ESI) m/z [M + H] 553.14. ^1^H-NMR δ (ppm): 1.22 (s, 2H, Pp-CH_2_-CH_2_-CH_2_), 1.37 (s, 6H, 2 × 5-CH_3_), 1.57–1.72 (m, 4H, Pp-CH_2_-CH_2_, N1-CH_2_-CH_2_), 3.07–3.13 (m, 6H, Pp-CH_2_, Pp-2,6-H), 3.24–3.29 (t def., 2H, N1-CH_2_), 3.37–3.55 (m, 4H, Pp-3,5-H), 4.60 (s, 2H, N3-CH_2_), 7.00–7.05 (m, 1H, Pp-Ph-6-H), 7.15–7.17 (d def., 2H, Ph-6-H, Pp-Ph-5-H), 7.21–7.29 (m, 1H, Pp-Ph-3-H), 7.39–7.43 (m, 1H, Ph-5-H), 7.63–7.64 (d def., 1H, Ph-3-H). IR (KBr) (cm^−1^): 3463 (NH), 3050 (CH Ar), 2987 (CH), 2359 (NH^+^), 1768(C2=O), 1705(C4=O), 1447 (C=C Ar).

#### 4.1.5. General Procedure to Obtain Final Products (**12**–**15**)

Piperazine derivatives (3 mmol) and the 1-oxiran-2-ylmethyl derivative **24** (3 mmol) were dissolved in acetone (15–20 mL). Mixture was evaporated and the solvent-free residue was irradiated in household microwave oven (300–450 W) for 3–7 min, under TLC control. The resulted glassy mass was dissolved in anhydrous ethanol and saturated with gaseous HCl to give a pure product in the hydrochloride form (Method A). When Method A did not provide a precipitate of an expected hydrochloric salt, the following procedure was further involved (Method B): the solution (from Method A) was evaporated, a residue dissolved in anhydrous ethanol. The cold diethyl ether (0 °C) was added until a precipitation appeared, followed by mixing in a cork flask for 30 min. The mixture was cooled at <−10 °C for 48–168 h, filtered off and the precipitate washed with 2–5 mL of cold ether or acetone to give the desirable product.

##### 3-(2,4-Dichlorobenzyl)-1-(3-(4-(2-fluorophenyl)piperazin-1-yl)-2-hydroxypropyl)-5,5-dimethylimidazolidine-2,4-dione hydrochloride (**12**)

1-(2-Fluorophenyl)piperazine (3 mmol, 0.54 g) and the 1-oxiran-2-ylmethyl derivative **24** (3 mmol, 1.03 g) were used. Parameters of electromagnetic radiation: 300 W for 2 min., 450 W for 1 min. and 450 W for 1 min. Product was obtained with Method B. White solid. Yield 61.0%; mp 99–101 °C. C_25_H_30_Cl_3_FN_4_O_3_ MW 558.14. LC/MS±: purity 98.76% t_R_ = 5.70, (ESI) m/z [M + H] 524.43. ^1^H-NMR δ (ppm): 1.40 (s, 6H, 2 × 5-CH_3_), 3.06–3.39 (m, 9H, Pp-3,5-H_a_H_1b_, Pp-2,6-H, Pp-CH_2_), 3.44–3.55 (m, 3H, N1-CH_2_, Pp-3,5-H_2b_), 3.67–3.69 (d def., 1H, CH-OH), 4.36–4.38 (d, J = 7.7 Hz, 1H, OH), 4.61 (s, 2H, N3-CH_2_), 6.99–7.25 (m, 5H, Pp-Ph-3,4,5,6-H, Ph-6-H), 7.39–7.42 (m, 1H, Ph-5-H), 7.62–7.63 (d def., 1H, Ph-3-H), 10.69 (br. s, 1H, NH^+^).

##### 3-(2,4-Dichlorobenzyl)-1-(3-(4-(4-fluorophenyl)piperazin-1-yl)-2-hydroxypropyl)-5,5-dimethylimidazolidine-2,4-dione hydrochloride (**13**)

1-(4-Fluorophenyl)piperazine (3 mmol, 0.54 g) and the 1-oxiran-2-ylmethyl derivative **24** (3 mmol, 1.03 g) were used. Parameters of electromagnetic radiation: 300 W for 3 min. Product was obtained with Method B. White solid. Yield 90.0%; mp 186–188 °C. C_25_H_30_Cl_3_FN_4_O_3_ MW 558.14. LC/MS±: purity 100.0% t_R_ = 5.57, (ESI) m/z [M + H] 524.43. ^1^H-NMR δ (ppm): 1.40 (s, 6H, 2 × 5-CH_3_), 3.08–3.34 (m, 10 H, Pp-2,3,5,6-H, Pp-CH_2_), 3.68 (s, 3H, CH-OH, N1-CH_2_), 4.36 (s, 1H, OH), 4.61 (s, 2H, N3-CH_2_), 6.97–7.11 (m, 4H, Pp-Ph-2,3,5,6-H), 7.22–7.25 (d, 1H, Ph-6-H), 7.39–7.42 (m, 1H, Ph-5-H), 7.62–7.63 (d, J = 1.8 Hz, 1H, Ph-3-H), 10.62 (br. s, 1H, NH^+^).

##### 3-(2,4-Dichlorobenzyl)-1-(3-(4-(2,4-difluorophenyl)piperazin-1-yl)-2-hydroxypropyl)-5,5-dimethylimidazolidine-2,4-dione hydrochloride (**14**)

1-(2,4-Difluorophenyl)piperazine (3 mmol, 0.59 g) and the 1-oxiran-2-ylmethyl derivative **24** (3 mmol, 1.03 g) were used. Parameters of electromagnetic radiation: 300 W for 3 min. Product was obtained with Method B. White solid. Yield 37.0%; mp 179–181 °C. C_25_H_29_Cl_3_F_2_N_4_O_3_ MW 576.13. LC/MS±: purity 98.42% t_R_ = 5.63, (ESI) m/z [M + H] 542.42. ^1^H-NMR δ (ppm): 1.40 (s, 6H, 2 × 5-CH_3_), 3.09–3.24 (m, 6H, Pp-2,6-H, Pp-CH_2_), 3.34–3.38 (m, 4H, Pp-3,5-H), 3.51–3.65 (m, 3H, CH-OH, N1-CH_2_), 4.34 (s, 1H, OH), 4.62 (s, 2H, N3-CH_2_), 6.98–7.20 (m, 2H, Pp-Ph-3,5-H), 7.22–7.27 (d, 2H, Ph-6-H, Pp-Ph-6-H), 7.39–7.42 (m, 1H, Ph-5-H), 7.62–7.63 (d, J = 2.0 Hz, 1H, Ph-3-H), 10.52 (br. s, 1H, NH^+^).

##### 3-(2,4-Dichlorobenzyl)-1-(3-(4-benzhydrylpiperazin-1-yl)-2-hydroxypropyl)-5,5-dimethylimidazolidine-2,4-dione hydrochloride (**15**)

1-(Diphenylmethyl)piperazine (3 mmol, 0.882 g) and the 1-oxiran-2-ylmethyl derivative **24** (3 mmol, 1.03 g) were used. Parameters of electromagnetic radiation: 450 W for 2 min, then 300 W for 1 min and 450 W for 4 min. Product was obtained with Method A. White solid. Yield 74.0%; mp 147–149 °C. C_32_H_37_Cl_3_N_4_O_3_ MW 630.19. LC/MS±: purity 100.0% t_R_ = 6.47, (ESI) m/z [M + H] 596.56. ^1^H-NMR δ (ppm): 1.38 (s, 6H, 2 × 5-CH_3_), 2.37–2.45 (m, 2H, Pp-CH_2_), 2.76–2.80 (d def., 2H, Pp-2,6-H_a_), 2.96–3.53 (m, 9H, Pp-2,6-H_b_, Pp-3,5-H, CH-OH, N1-CH_2_), 4.27 (br. s, 1H, OH), 4.43 (s, 1H, CH(Ph)_2_), 4.60 (s, 2H, N3-CH_2_), 7.17–7.24 (m, 3H, Pp-Ph-5,5′-H, Ph-6-H), 7.27–7.32 (t def., 4H, Pp-Ph-3,4,3′,4′-H)), 7.38–7.43 (m, 5H, Pp-Ph-2,6,2′,6′-H, Ph-5-H), 7.62–7.63 (d, J = 2.0 Hz, 1H, Ph-3-H), 10.33 (br. s, 1H, NH^+^).

### 4.2. Crystallographic Studies

Crystals suitable for an X-ray structure analysis were grown from butyl acetate for **6** and acetonitryl for **15**, by slow evaporation of the solvent at room temperature.

Data for single crystals of **6** were collected at 100 K on the Bruker-Nonius Kappa CCD four circle diffractometer equipped with a Mo (0.71069 Å) Kα radiation source, while for single crystals of **15** were collected at 130 K using the Oxford Diffraction SuperNova four circle diffractometer equipped with the Mo (0.71073 Å) Kα radiation source, graphite monochromator. Positions of all non-hydrogen atoms were determined by direct methods using SHELXS-97 [22] for **6** and SIR-2014 for **15** [23] programs. Refinement and further calculations were carried out using SHELXL-2018 program [24]. All non-hydrogen atoms were refined anisotropically using weighted full-matrix least-squares on F^2^. The hydrogen atoms attached to nitrogen and oxygen atoms were identified on difference Fourier maps, whereas all hydrogen atoms bonded to carbon atoms were included in the structure at idealized positions and were refined using a riding model with U_iso_(H) fixed at 1.2 U_eq_ of C and 1.5 U_eq_ for methyl groups. For molecular graphics, MERCURY [25] program was used.

Compound **6**: C_27_H_35_F_2_ O_2_N_4_^+^ Cl^−^, M_r_ = 521.04, crystal size = 0.24 × 0.26 × 0.38 mm^3^, monoclinic, space group P2_1_/c, a = 13.5070(3) Å, b = 8.1930(2) Å, c = 26.3923(6) Å, β = 113.387(2)°, V = 2680.7(1) Å^3^, Z = 4, T = 100(2) K, 13924 reflections collected, 5882 unique reflections (R_int_ = 0.0251), R1 = 0.0376, wR2 = 0.0877 [I > 2σ(I)] and R1 = 0.0475, wR2 = 0.0940 [all data].

Compound **15**: C_32_H_37_Cl_2_O_3_N_4_^+^ Cl^−^, M_r_ = 632.00, crystal size = 0.07 × 0.24 × 0.41 mm^3^, monoclinic, space group P2_1_/c, a = 7.3343(2) Å, b = 16.1643(3) Å, c = 28.7674(6) Å, β = 110.934(2)°, V = 3185.4(1) Å^3^, Z = 4, T = 130(2) K, 42439 reflections collected, 6171 unique reflections (R_int_ = 0.0795), R1 = 0.0447, wR2 = 0.1152 [I > 2σ(I)] and R1 = 0.0558, wR2 = 0.1262 [all data].

CCDC 2015564-2015565 contain the supplementary crystallographic data. These data can be obtained free of charge from The Cambridge Crystallographic Data Centre via www.ccdc.cam.ac.uk/data_request/cif.

### 4.3. Microbiological Studies

*S. epidermidis* K/14/1345 was isolated from the blood of a patient admitted to University Children’s Hospital in Krakow, Prokocim. *S. epidermidis* ATCC 12228 was purchased from GRASO Biotech (Poland). Bacteria were maintained and grown on Trypticase Soy agar supplemented with 5% sheep blood (TSA II; Becton Dickinson, Germany). Cation-adjusted Mueller-Hinton (MH II) broth and Tryptic Soy broth (TSB), used in susceptibility testing and EtBr accumulation assays, were obtained from Difco, Becton Dickinson (Spain) and bioMérieux (France), respectively. The culture media were prepared by dissolving appropriate dry culture media powders in distilled water. The liquid media obtained were sterilized by autoclaving at 121 °C for 15 min. Compounds tested and erythromycin (Sigma-Aldrich Chemie GmbH, Darmstadt, Germany) used in microbiological assays were dissolved in dimethyl sulfoxide (DMSO; Merck, Germany) and 70% ethanol, respectively. EtBr, which was used to evaluate the efflux pump inhibitory activity of compounds tested, was obtained from Sigma-Aldrich Chemie GmbH (Munich, Germany). Stock solution of the dye at 2 µg/mL was freshly prepared in phosphate buffered saline (PBS) with 0.4% glucose (Sigma-Aldrich Quimica, Madrid, Spain) before use. PBS in tablets (0.01 M phosphate buffer, 0.0027 M potassium chloride and 0.137 M sodium chloride, pH 7.4) was dissolved in distilled, sterile water and autoclaved. Glucose (Chempur, Poland) was dissolved in distilled, sterile water and stored at 4 °C. 

#### 4.3.1. Susceptibility Testing 

Susceptibility testing was carried out according to the standard microdilution method in MH II broth following the guidelines provided by the Clinical and Laboratory Standards Institute (CLSI) [26]. MIC values were detected through the measurement of optical density at 600 nm in Tecan Sunrise reader. 

Initially, minimal inhibitory concentration (MIC) values of 15 phenylpiperazine 5,5-dimethylhydantoin derivatives were determined against two strains of *S. epidermidis*, the reference strain ATCC 12228 and the clinical isolate K/14/1345 whose overexpression of Msr(A) transporter is associated with MS_B_ phenotype. Next, the adjuvant-like effect of 5,5-dimethylhydantoins was evaluated by the determination of erythromycin MICs in the absence and in the presence of compounds tested. The concentrations of compounds employed in the MIC reduction assay were not greater than 1/2 of their MIC values, therefore, cell viability was not influenced by the intrinsic antibacterial activity of these compounds. The final concentration of DMSO in broth medium never exceeded 2.5% (*v*/*v*) and did not affect bacterial viability, which was confirmed by conducting additional assays. The MIC values of 5,5-dimethylhydantoins and erythromycin were recorded as the lowest concentrations of compounds/antibiotic at which no growth of bacteria was detected (OD_600_ < 0.07) after 18-h incubation at 37 °C [26]. The results obtained in the MIC reduction assay were expressed by employing activity gain parameter (A) calculated as the MIC of antibiotic tested alone to its MIC tested in combination with compound examined (Equation (1)) [21,27]. All MIC evaluations were performed in at least two independent experiments.

(1) $$A\;=\;(\frac{MIC_{ant}}{MIC_{ant}\;+\;MIC_{cpd}})$$

#### 4.3.2. Ethidium Bromide Accumulation Assay

The methodology is commonly used for the expedite detection and qualification of the efflux capabilities in bacteria as well as for the search of new efflux pump inhibitors that could overcome bacterial drug resistance. It enables direct detection of EtBr accumulation inside bacterial cells on a real-time basis following the monitoring of the fluorescence of the dye over a period of time [28,29]. EtBr has been shown to be particularly suitable for this method since it fluoresces weakly in aqueous solution (extracellularly) and becomes strongly fluorescent in the hydrophobic environment, especially when it penetrates the bacterial cell wall and binds to cellular components such as DNA. Consequently, the lower is the level of efflux of EtBr, the higher is the fluorescence of this dye within bacterial cells [20,30]. The EtBr accumulation assay is performed under conditions that promote efflux, i.e., the presence of a source of energy (glucose) and the temperature of 37 °C. The results obtained in the experiment are illustrated by the comparison of accumulation of EtBr in the absence and presence of compounds tested in efflux pump overexpressing strain. The changes in the fluorescence of EtBr are measured at excitation and emission wavelengths of 530 and 585 nm, respectively [29,30]. Prior to the main experiment, MICs of EtBr against *S. epidermidis* strain K/14/1345 which overexpresses Msr(A) efflux pump as well as in the reference strain *S. epidermidis* ATCC 12228 were evaluated according to the protocol described above. Moreover, the optimal concentration of EtBr that was within the capability of the bacteria to extrude from the cytoplasm was established. For the main accumulation assay in which the EPI properties of compounds were determined, bacteria were grown in TSB at 37 °C with shaking at 150 rpm until they reached an OD_600_ of 0.6. Bacterial cultures were then centrifuged at 12,500× *g* for 3 min, the supernatant was discarded and the remaining pellet washed twice in PBS. Thereafter, the pellet was resuspended in PBS pH 7.4 containing 0.4% glucose and the resulting bacterial suspension was adjusted to OD_600_ = 0.6. Forty-five microliters of bacterial suspension were treated with 1 μg/mL EtBr and compounds tested at the concentration of 1/4 and 1/2 of their MICs. The level of EtBr accumulation was established using a microplate reader (EnSpire, PerkinElmer, Waltham, MA, USA), with an excitation wavelength of λ_ex_ = 530 nm and an emission wavelength of λ_em_ = 585 nm at a constant temperature of 37 °C for 30 min. The modulatory activity of compounds on the efflux pump of interest was evaluated in at least three independent experiments (biological replicates). The results were presented on graphs using GraphPad Prism 5.0 (GraphPad Software Inc., San Diego, CA, USA). Each value of the experimental accumulation curve was corrected by subtracting the fluorescence of the background corresponding to the initial EtBr concentration in PBS. To compare the inhibitory activity of the potent EPIs against overexpressed Msr(A) exporter, the relative fluorescence index (RFI) and the specific activity (SA) of compounds on the accumulation of EtBr by *S. epidermidis* K/14/1345 clinical isolate were calculated. The modulatory effect of the compounds towards transporter protein in *S. epidermidis* K/14/1345 was compared with the one determined in the reference *S. epidermidis* strain ATCC 12228. The RFI parameter was calculated using the relative final fluorescence (RFF) of the last time point (minute 20) of the EtBr accumulation curve according to the following Equations (2) and (3) [17]:

(2) $$\mathrm{RFF}\;={\mathrm{RF}}_{\mathrm{treated}}-{\mathrm{RF}}_{\mathrm{untreated}}$$

(3) $$\mathrm{RFI}\;=\;\frac{\mathrm{RFF}}{{\mathrm{RF}}_{\mathrm{untreated}}}$$

where RF_treated_ corresponds to the relative fluorescence at the last time point of the EtBr retention curve in the presence of a compound tested and RF_untreated_ is the relative fluorescence at the last time point of the EtBr retention curve in the absence of a compound tested.

Dividing RFI value by the number of micromoles of 5,5-dimethylhydantoin derivative used in the assay provided a specific activity of inhibition of the Msr(A) efflux pump present in *S. epidermidis* bacterium (Equation (4)) [17].

(4) $$\mathrm{SA}\;=\;\frac{\mathrm{RFI}}{n\;\left[µ\mathrm{mol}\right]}$$

## 5. Conclusions

Herein, we identified first synthetic compounds able to inhibit Msr(A) efflux pump transporter present in *S. epidermidis* bacterium. Design, chemical synthesis, comprehensive microbiological assays and X-ray supported deeper insight into structural properties of two compounds with different activity. As results, five compounds with significant potency to inhibit the dye-substrate of the ABC transporter, Msr(A), were found. This finding may contribute to form the basis for further search for potent EPIs among hydantoin derivatives in order to enhance their inhibitory activity towards Msr(A) efflux pump system. The most active EPI identified in this study, i.e., 3-(2,4-dichlorobenzyl)-1-(5-(4-(2-fluorophenyl)piperazin-1-yl)pentyl) derivative of 5,5-dimethylhydantoin (**9**), seems to be a good lead structure for further pharmacomodulations. In a therapeutic context, this could open new perspectives for rational design and synthesis of antimicrobial resistance breakers that are able to restore the activity of macrolide antibiotics against *S. epidermidis* strains, in the case of the membrane-associated resistance mechanisms.

## Figures and Tables

**Table 1:**
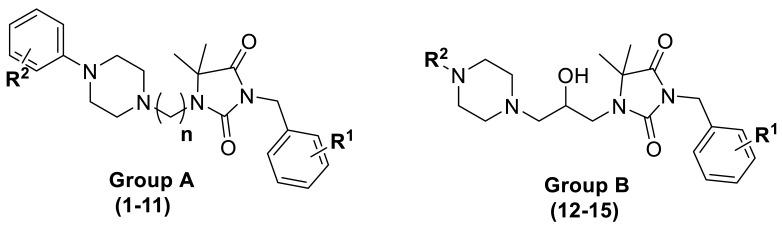
Structures of phenylpiperazine 5,5- dimethylhydantoin derivatives (**1**–**15**) investigated in the study.

Cpd	Group	R^1^	n	R^2^
**1**	A	H	5	2,4-diF
**2**	A	H	5	H
**3**	A	H	5	2-MeO
**4**	A	H	5	3-MeO
**5**	A	H	5	2-F
**6**	A	4-F	5	4-F
**7**	A	4-F	5	2,4-diF
**8**	A	4-Cl	6	2-F
**9**	A	2,4-diCl	5	2-F
**10**	A	2,4-diCl	5	4-F
**11**	A	2,4-diCl	5	2,4-diF
**12**	B	2,4-diCl	-	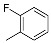
**13**	B	2,4-diCl	-	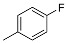
**14**	B	2,4-diCl	-	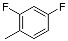
**15**	B	2,4-diCl	-	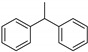

**Table 2:** Parameters of intermolecular interactions for **6** and **15**.

Cmp.	Interaction	H···A (Å)	D···A (Å)	D-H···A (°)	Symmetry Code
**6**	N2-H2N···Cl1	2.14	3.023(1)	176	
	C14-H14A···Cl1	2.72	3.558(1)	142	−x + 1, −y + 1, −z + 1
	C20-H20···Cl1	2.85	3.753(1)	159	−x + 1, −y + 1, −z + 1
	C28-H28C···Cl1	2.84	3.735(2)	152	−x, −y + 1, −z + 1
	C10-H10A···O2	2.36	3.187(2)	141	−x, −y + 1, −z + 1
	C12-H12B···O4	2.50	3.046(2)	114	x + 1, −y + 1/2, z + 1/2
	C21-H21B···O4	2.66	3.385 (2)	131	−x − 1, y + 1/2, −z + 1/2
	C13-H13B···N4	2.57	3.531(2)	163	−x + 1, −y, −z + 1
	C29-H29C···F1	2.54	3.500(2)	166	−x + 1, −y, −z + 1
**15**	N2-H2N···Cl1	2.07	3.067(2)	174	
	O1-H1···Cl1	2.12	3.049(2)	168	
	C9-H9A···Cl1	2.66	3.645(2)	179	X − 1, y, z
	C16-H16···Cl1	2.91	3.638(2)	135	X − 1, y, z
	C21-H21C···Cl1	2.95	3.755(2)	140	−x + 2, −y + 1, −z + 1
	C6-H6B···O4	2.59	3.550(3)	163	x + 1, y, z
	C9-H9A···O1	2.36	3.325(3)	166	−x + 2, −y + 1, −z + 1
	C18-H18···O2	2.58	3.463(3)	156	−x + 1, −y + 2, −z + 1
	C21-H21A···O1	2.64	3.358(3)	131	X − 1, y, z

**Table 3:** Antibacterial activity of phenylpiperazine 5,5-dimethylhydantoin derivatives and erythromycin against *S. epidermidis* K/14/1345 and *S. epidermidis* ATCC 12228 strains.

Compounds	MIC [µM] (µg/mL)	
	*S. epidermidis* K/14/1345	*S. epidermidis* ATCC 12228
**1**	>250 (130.26) ^1^	250 (130.26)
**2**	>250 (121.27) ^1^	>250 (121.27) ^1^
**3**	1000 (515.09)	250 (128.77)
**4**	>125 (64.39) ^1^	62.5 (32.19)
**5**	>500 (251.53) ^1^	250 (125.96)
**6**	>250 (130.26) ^1^	250 (130.26)
**7**	125 (67.38)	250 (134.76)
**8**	>62.5 (34.38) ^1^	62.5 (34.38)
**9**	>62.5 (35.74) ^1^	31.25 (17.88)
**10**	>31.25 (17.88) ^1^	31.25 (17.88)
**11**	>31.25 (18.44) ^1^	15.63 (9.22)
**12**	>125 (70) ^1^	62.5 (35)
**13**	>125 (69.99) ^1^	125 (69.99)
**14**	>125 (72.24) ^1^	62.5 (36.12)
**15**	>31.25 (19.76) ^1^	7.81 (4.94)
**Erythromycin**	0.087 (64)	0.00068 (0.5)

^1^ The highest possible concentration of a compound (due to precipitation) at which growth of bacteria was observed.

**Table 4:** Specific activity (SA) of active 5,5-dimethylhydantoin derivatives tested at the concentration corresponding to 1/2 and 1/4 of their MIC values on the accumulation of EtBr in *S. epidermidis* strain K/14/1345 and ATCC 12228.

SA			SA		
*S. epidermidis* K/14/1345			*S. epidermidis* ATCC 12228		
	RFI/µmol			RFI/µmol	
Compound	1/2 MIC ^1^	1/4 MIC ^1^	Compound	1/2 MIC	1/4 MIC
**1**	12.22	13.31	**1**	1.48	0.094
**6**	6.02	4.59	**6**	0.87	0.49
**7**	22.16	23.77	**7**	0.65	0.23
**9**	24.82	25.66	**9**	4.29	1.52
**14**	14.32	13.04	**14**	1.9	−5.27

^1^ The highest possible concentration that did not inhibit bacterial growth in case of precipitating compounds **1**, **6**, **9** and **14**.

## Supplementary Materials

The following are available online. Table S1: Influence of phenylpiperazine 5,5-dimethylhydantoin derivatives on the susceptibility of *S. epidermidis* strains to erythromycin, Figures S1a–S20a: Influence of different compounds on accumulation of EtBr in *S. epidermidis* K/14/1345 and *S. epidermidis* ATCC 12228. Figures S1b–S20b: Fluorescence intensity at last time point of the EtBr retention curve in the presence of different compounds.
