# Supplementary material for: Phenylpiperazine 5,5-Dimethylhydantoin Derivatives as First Synthetic Inhibitors of Msr(A) Efflux Pump in Staphylococcus epidermidis
Source: Molecules. 2020 Aug 20;25(17):3788. doi: 10.3390/molecules25173788 (PMC7503621; doi:10.3390/molecules25173788)
Supplement: Supplementary file 1 [file molecules-25-03788-s001.pdf]

Supplementary

Phenylpiperazine 5,5-dimethylhydantoin derivatives as first synthetic inhibitors of Msr(A) efflux pump in *Staphylococcus epidermidis*

Karolina Witek, Gniewomir Latacz, Ewa Żesławska, Aneta Kaczor, Joanna Czekajewska, Anna Chudzik, Elżbieta Karczewska, Wojciech Nitek, Katarzyna Kieć-Kononowicz, Jadwiga Handzlik

Chemistry

^{1}H NMRs

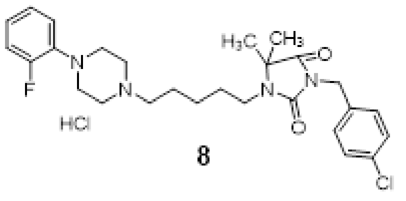
F
HCl
H_3C
CH_3
N
O
Cl

8

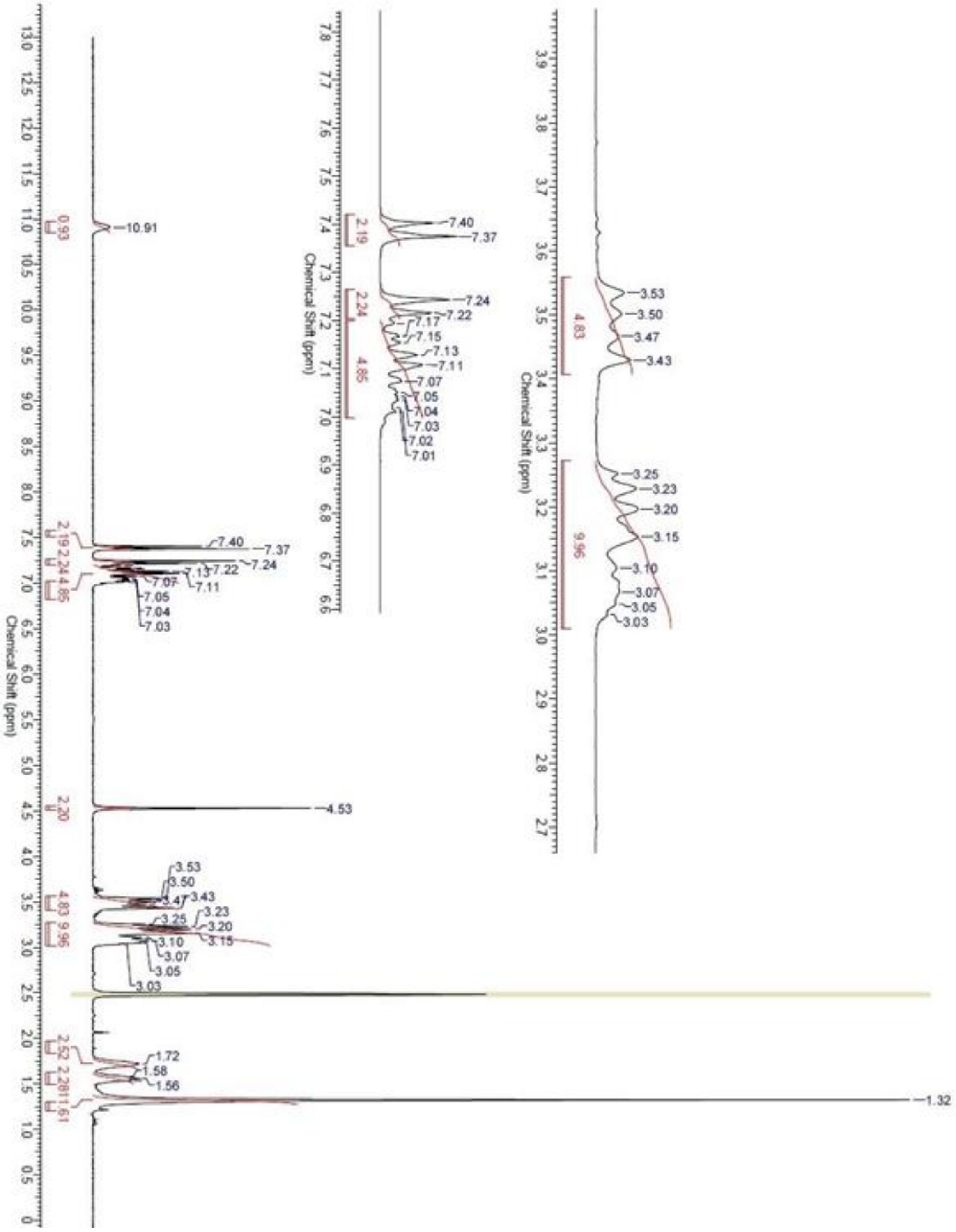
Chemical Shift (ppm)
10.91
7.40
7.37
7.24
7.22
7.13
7.11
7.07
7.05
7.04
7.03
4.53
3.53
3.50
3.47
3.43
3.25
3.23
3.20
3.15
3.10
3.07
3.05
3.03
1.72
1.58
1.56
1.32
0.93
2.19
2.24
4.85
2.20
4.83
9.96
2.52
2.28
11.61

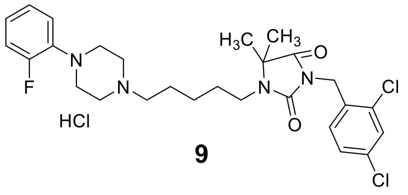

9

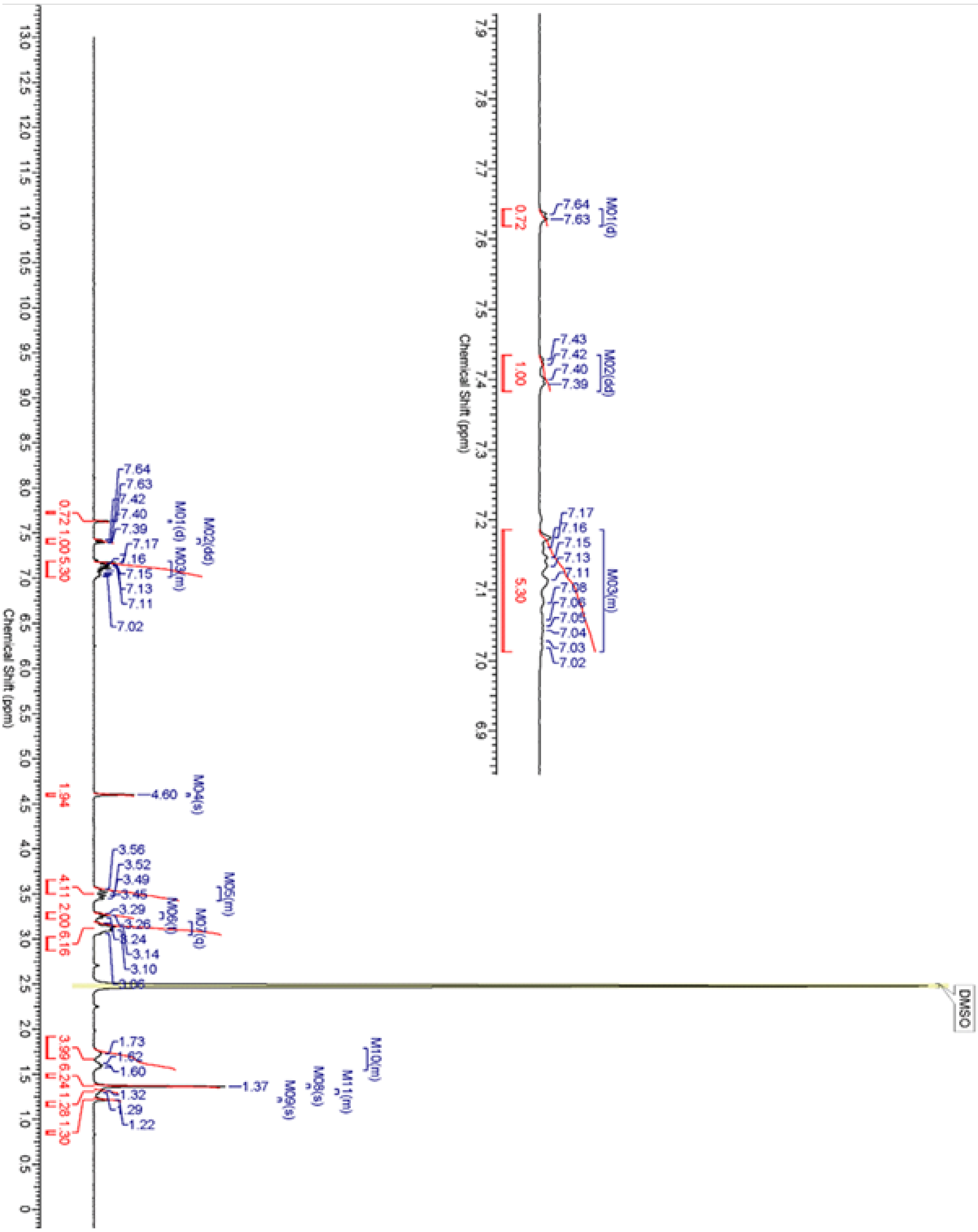

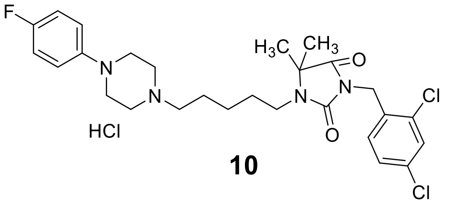
F
N
N
HCl
H_3C
CH_3
O
N
N
O
Cl
Cl

10

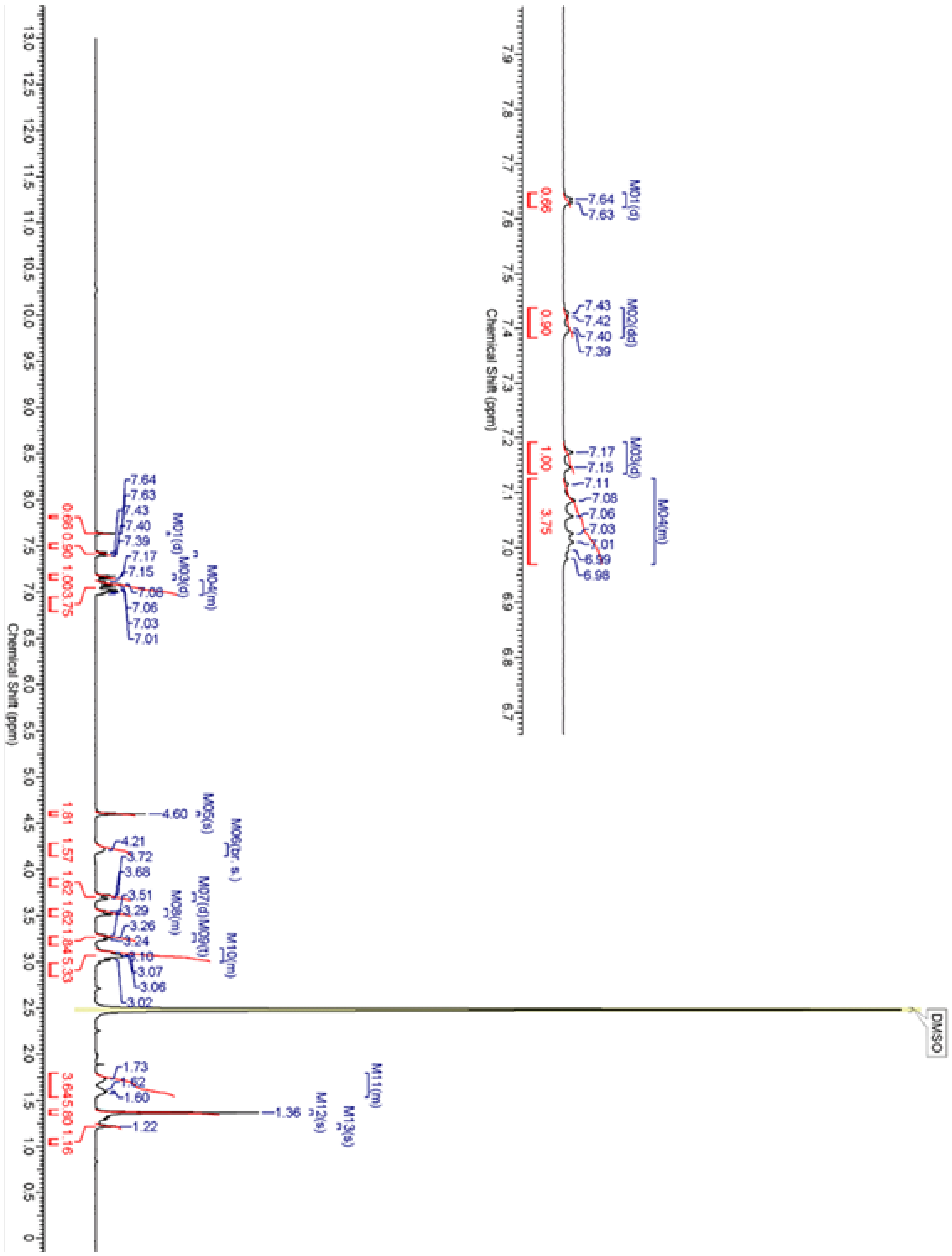
Chemical Shift (ppm)
DMSO
M01(d)
M02(dd)
M03(d)
M04(m)
M05(s)
M06(br. s.)
M07(d)
M08(m)
M09(t)
M10(m)
M11(m)
M12(s)
M13(s)

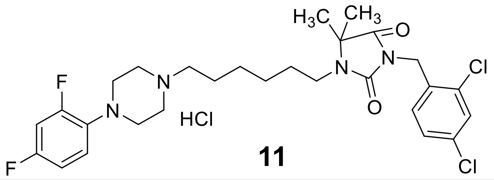

11

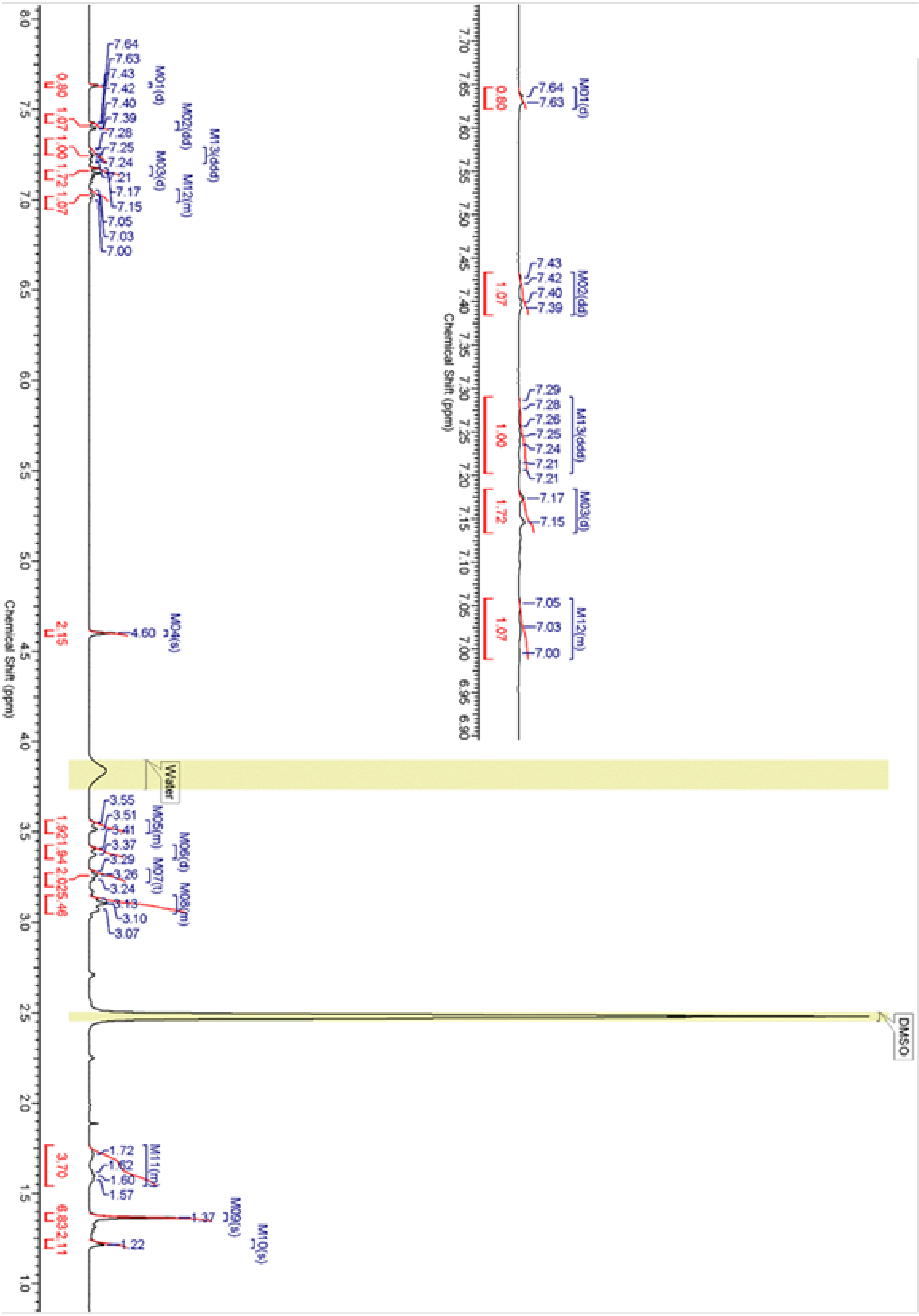

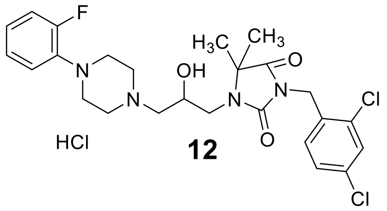

12

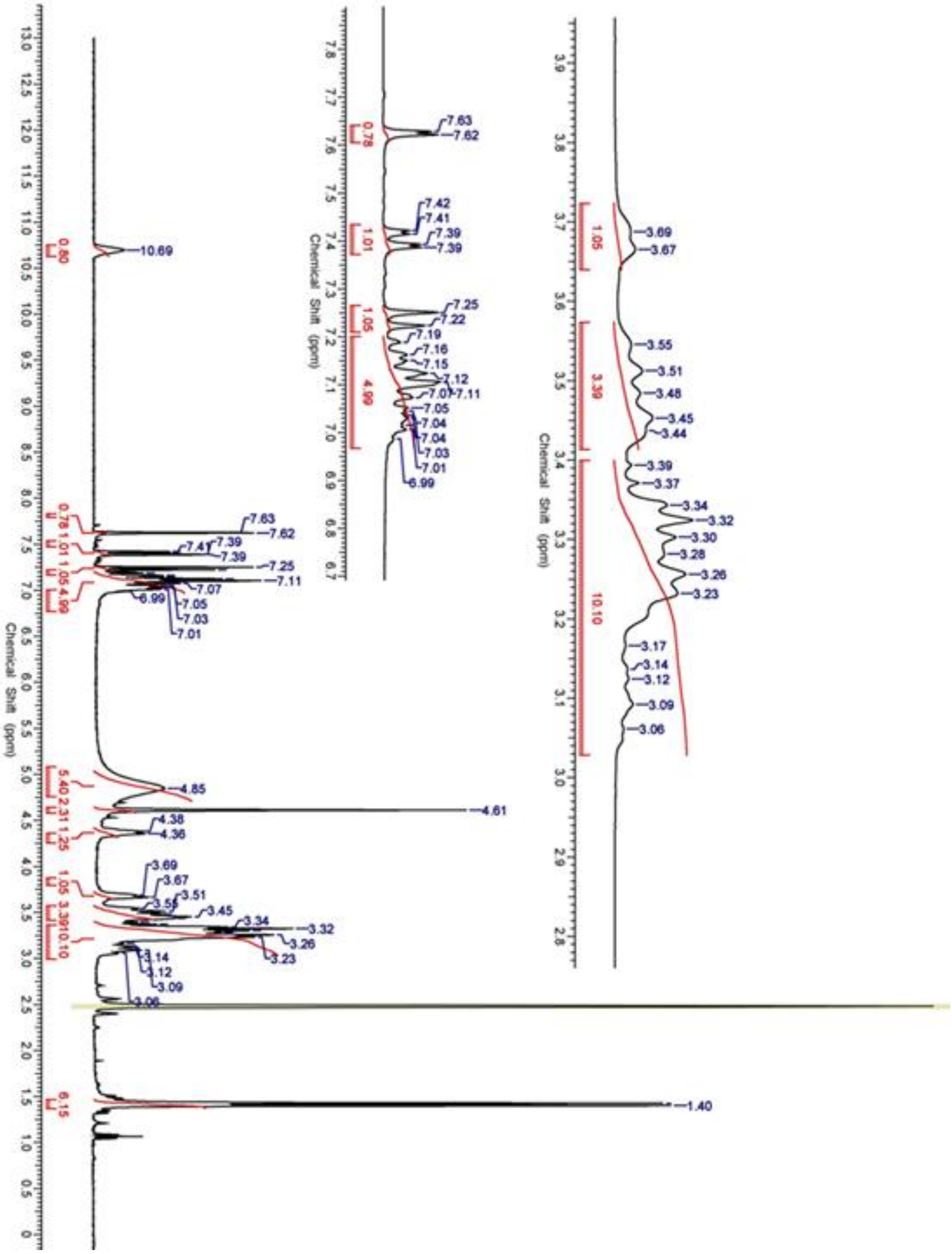

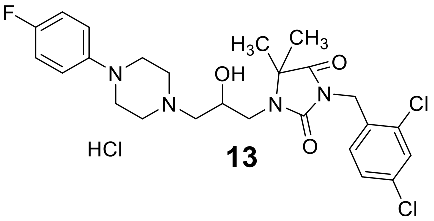
F
N
N
OH
N
N
O
O
Cl
Cl
HCl

13

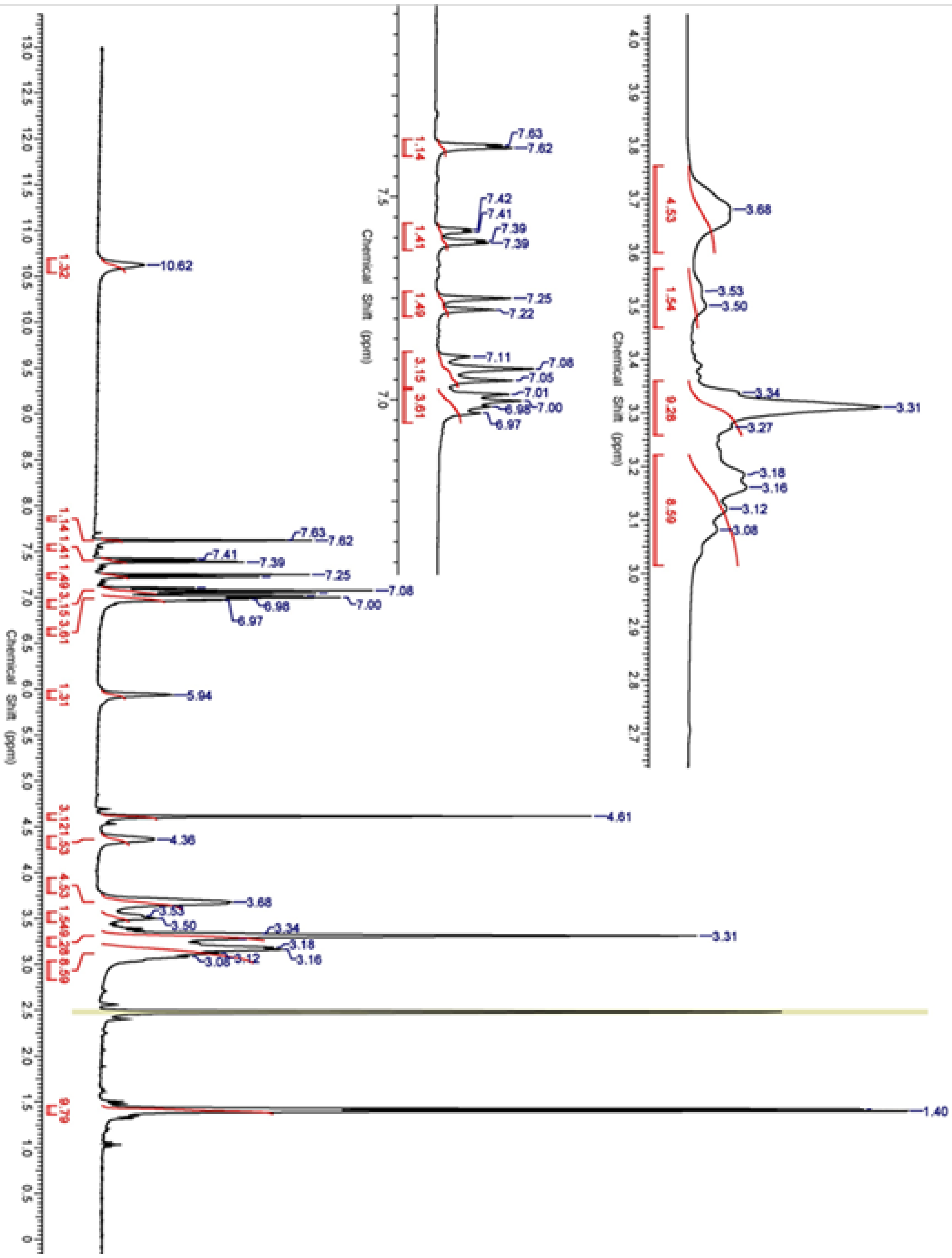
Chemical Shift (ppm)
10.62
7.63
7.62
7.41
7.39
7.25
7.08
7.00
6.98
6.97
5.94
4.61
4.36
3.68
3.53
3.50
3.34
3.31
3.18
3.16
3.12
3.08
1.40

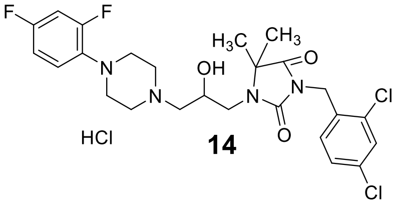

14

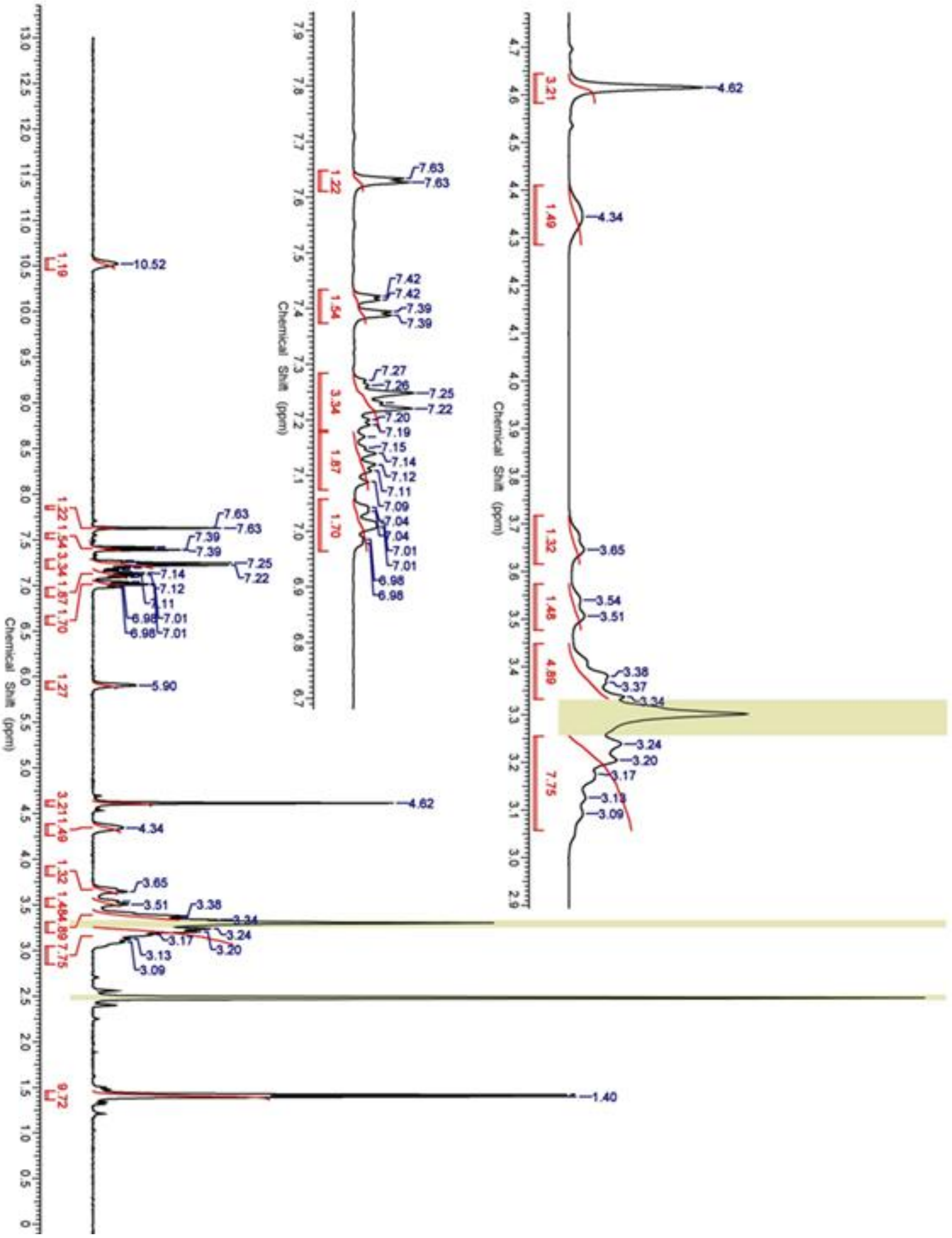

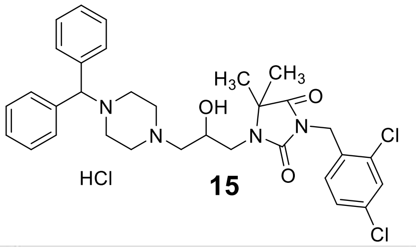

H_3C
CH_3
OH
HCl

15

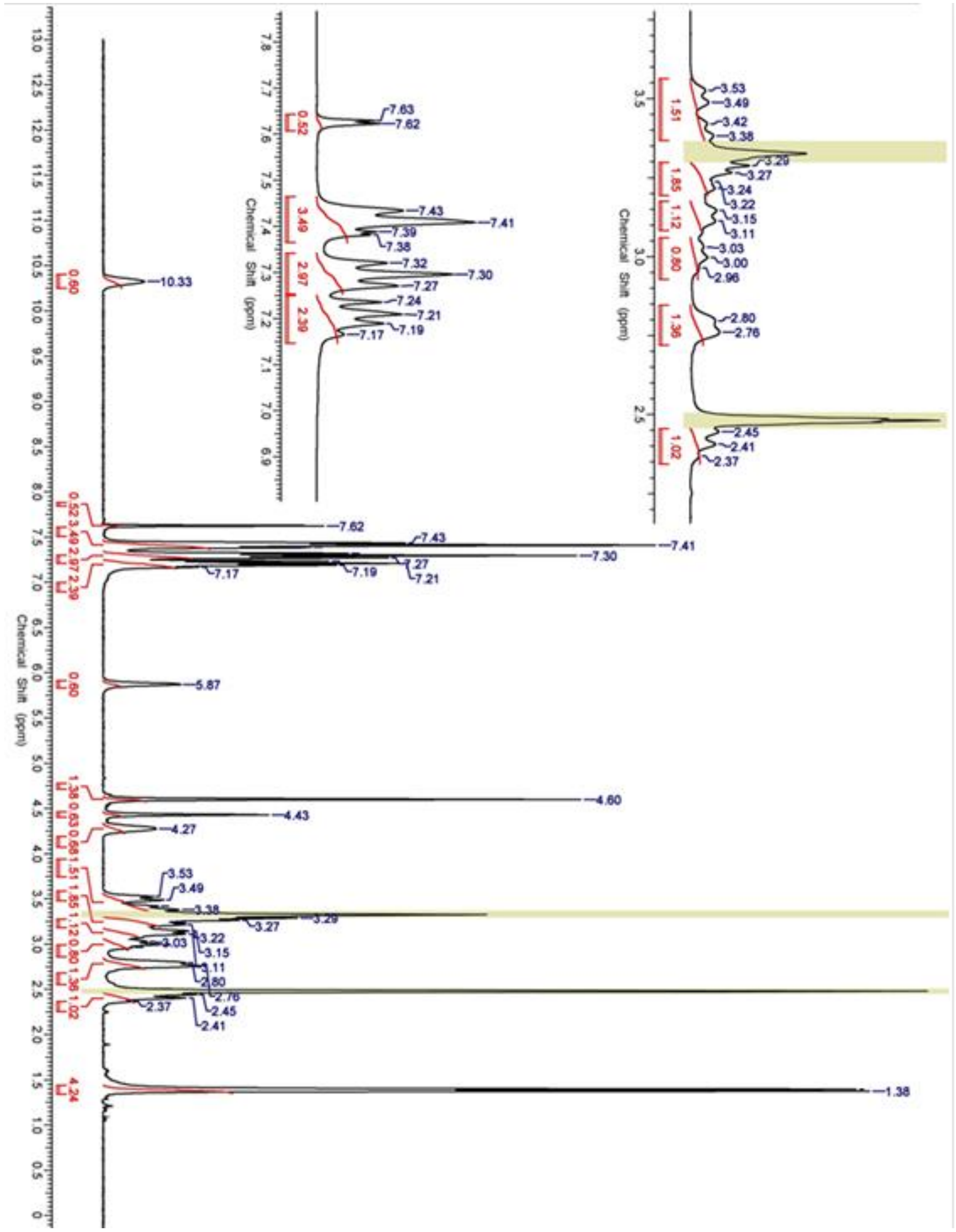

Chemical Shift (ppm)
10.33
7.62
7.43
7.41
7.30
7.27
7.21
7.19
7.17
5.87
4.60
4.43
4.27
3.53
3.49
3.38
3.29
3.27
3.22
3.15
3.11
3.03
2.80
2.76
2.45
2.41
2.37
1.38

Details of biological assays

Table S1. Effect of phenylpiperazine 5,5-dimethylhydantoin derivatives on the susceptibility of *S. epidermidis* strains to erythromycin.

Cpd	*S. epidermidis* K14/1345			*S. epidermidis* ATCC 12228		
	**Conc. of compound [μM]**	**Activity gain [A]**	**Range of reduction [μg/ml]**	**Conc. of compound [μM]**	**Activity gain [A]**	**Range of reduction [μg/ml]**
**1**	62.5	1	no effect	62.5	1	no effect
**2**	62.5	1	no effect	62.5	1	no effect
**3**	250	1	no effect	62.5	1	no effect
**4**	31.25	1	no effect	15.63	1	no effect
**5**	125	1	no effect	62.5	1	no effect
**6**	62.5	1	no effect	62.5	1	no effect
**7**	31.25	1	no effect	62.5	1	no effect
**8**	15.63	1	no effect	15.63	1	no effect
**9**	15.63	1	no effect	7.81	1	no effect
**10**	7.81	1	no effect	7.81	1	no effect
**11**	7.81	1	no effect	3.9	1	no effect
**12**	31.25	1	no effect	15.63	1	no effect
**13**	31.25	1	no effect	31.25	1	no effect
**14**	31.25	1	no effect	15.63	1	no effect
**15**	7.81	1	no effect	3.9	1	no effect

a)

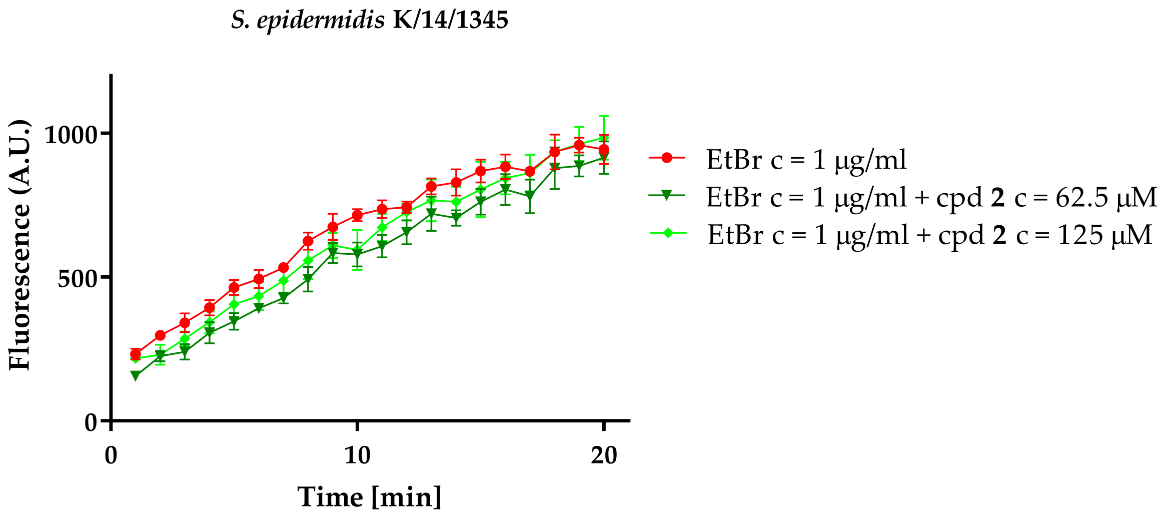

b)

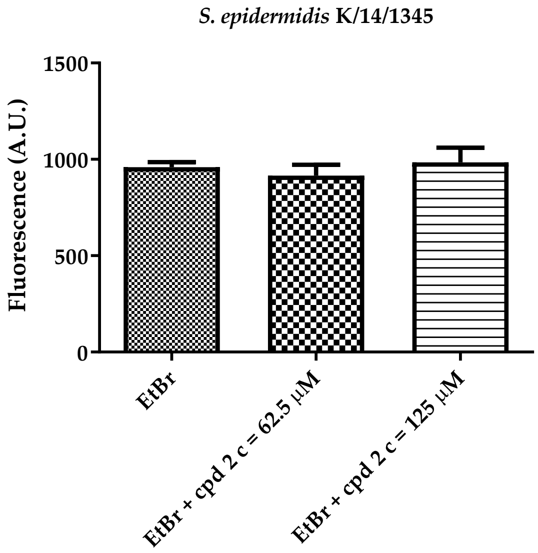

Figure 1S. a) Influence of compound **2** on accumulation of EtBr in *S. epidermidis* K/14/1345. Each data point expresses the mean ± standard deviation (SD) from four replicates; **b)** Fluorescence intensity at last (20 min) time point of the EtBr retention curve in presence of compound **2.** Statistical significance was calculated using a one-way analysis of ANOVA, followed by multiple comparison test ($p > 0.05$).

a)

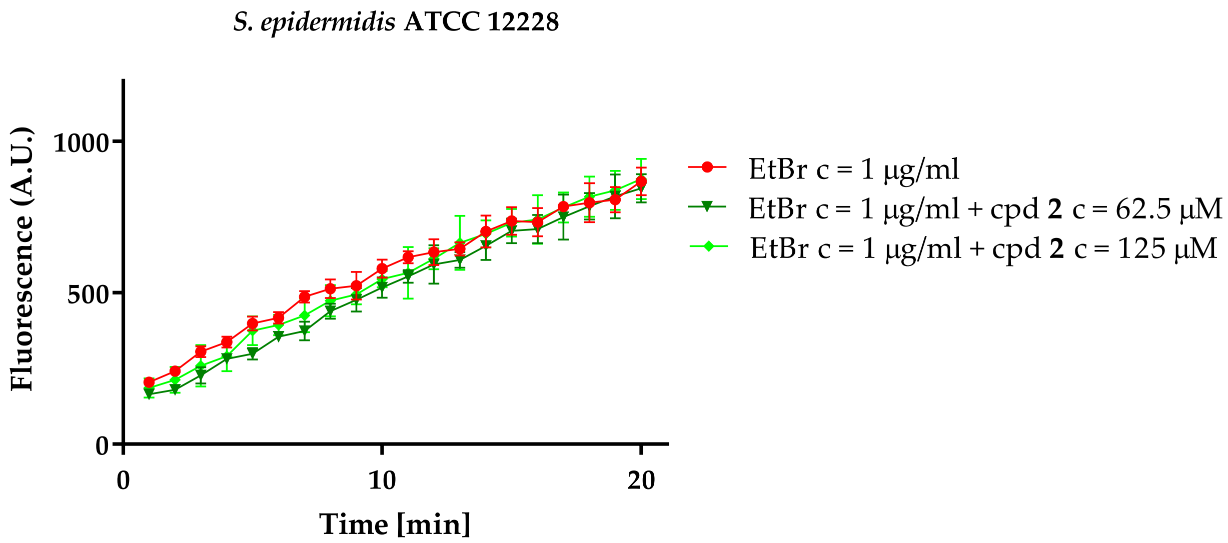

b)

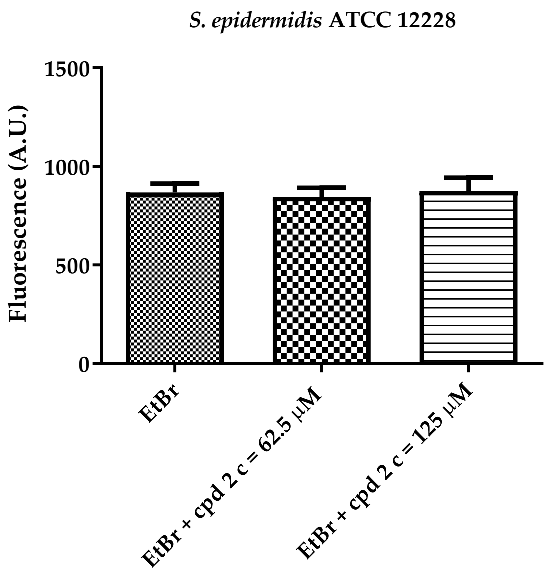

Figure 2S. a) Influence of compound **2** on accumulation of EtBr in *S. epidermidis* ATCC 12228. Each data point expresses the mean ± standard deviation (SD) from four replicates; **b)** Fluorescence intensity at 20 min time point of the EtBr retention curve in presence of **c**ompound **2.** Statistical significance was calculated using a one-way analysis of ANOVA, followed by multiple comparison test ($p > 0.05$).

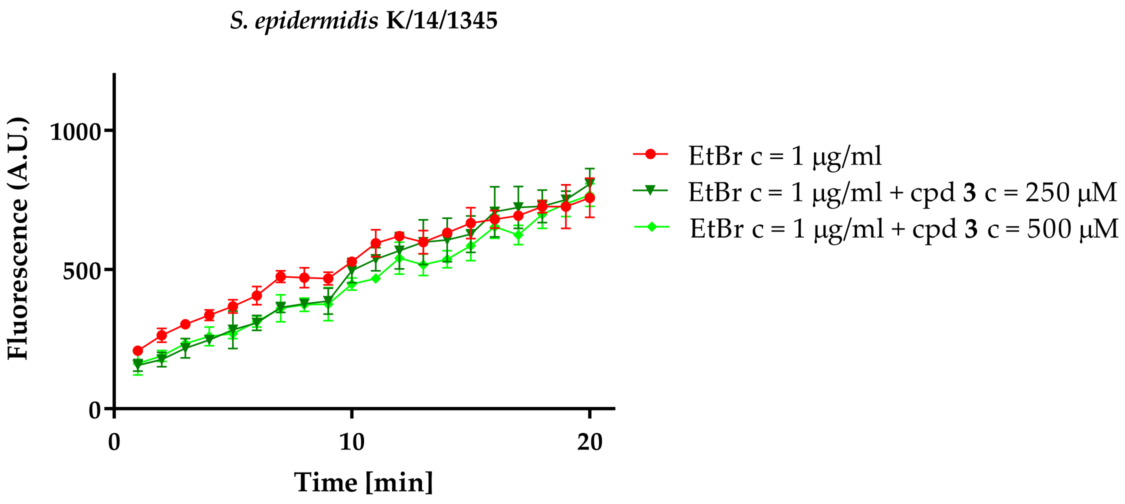

a)

b)

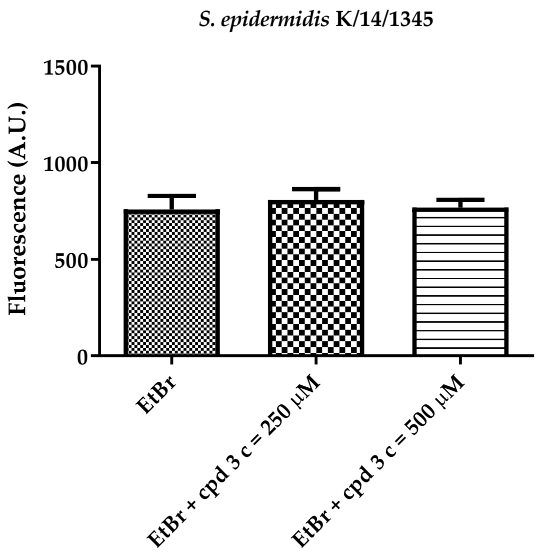

Figure 3S. a) Influence of compound **3** on accumulation of EtBr in *S. epidermidis* K14/1345. Each data point expresses the mean ± standard deviation (SD) from four replicates; **b)** Fluorescence intensity at 20 min time point of the EtBr retention curve in presence of compound **3.** Statistical significance was calculated using a one-way analysis of ANOVA, followed by multiple comparison test ($p > 0.05$).

a)

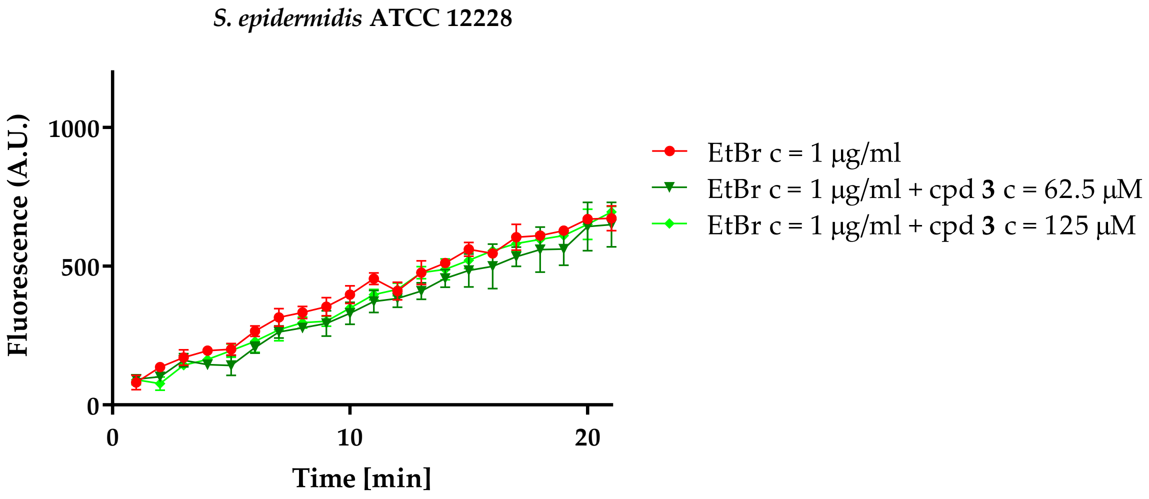

b)

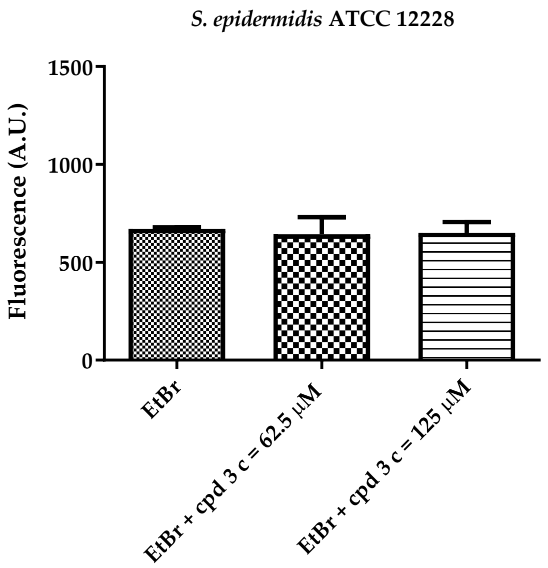

Figure 4S. a) Influence of compound **3** on accumulation of EtBr in *S. epidermidis* ATCC 12228. Each data point expresses the mean ± standard deviation (SD) from four replicates; **b)** Fluorescence intensity at 20 min time point of the EtBr retention curve in presence of compound **3.** Statistical significance was calculated using a one-way analysis of ANOVA, followed by multiple comparison test ($p > 0.05$).

a)

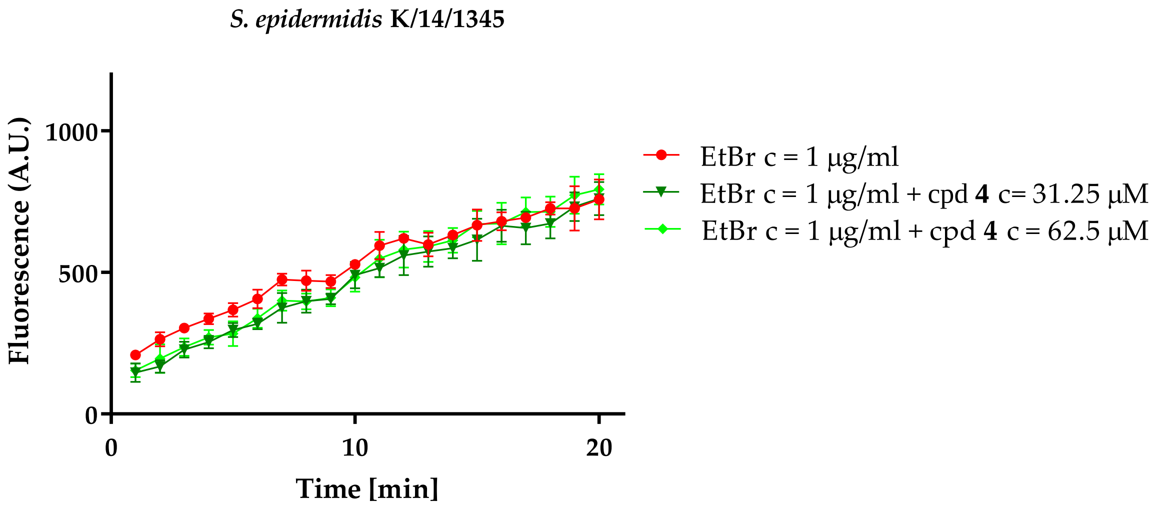

b)

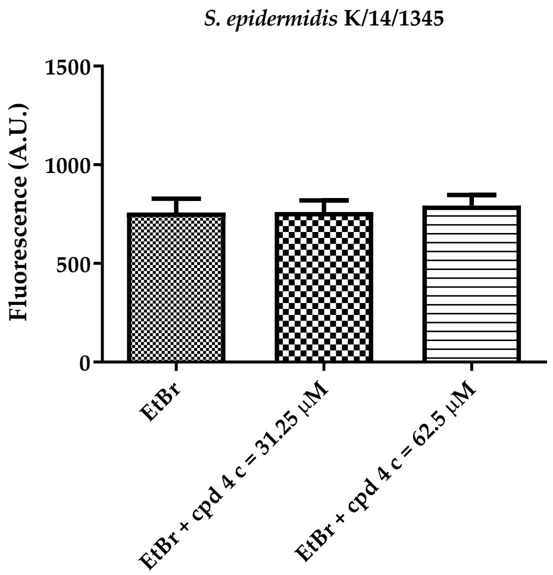

Figure 5S. a) Influence of compound **4** on accumulation of EtBr in *S. epidermidis* K/14/1345. Each data point expresses the mean ± standard deviation (SD) from four replicates; **b)** Fluorescence intensity at last (20 min) time point of the EtBr retention curve in presence of compound **4.** Statistical significance was calculated using a one-way analysis of ANOVA, followed by multiple comparison test ($p > 0.05$).

a)

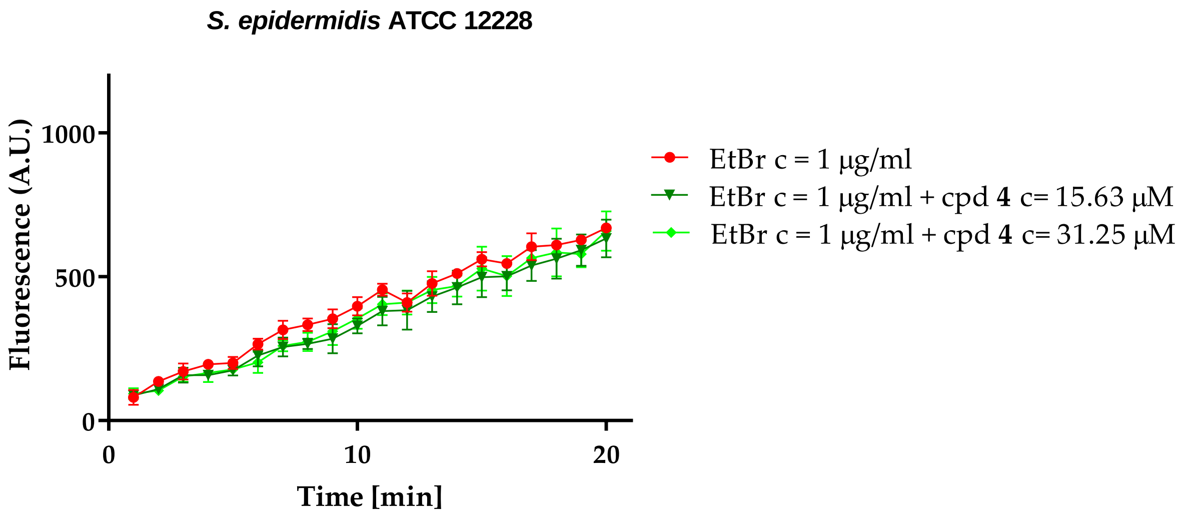

b)

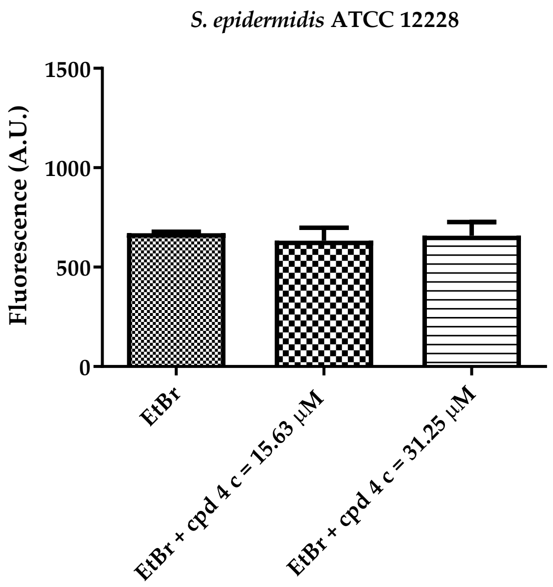

Figure 6S. a) Influence of compound **4** on accumulation of EtBr in *S. epidermidis* ATCC 12228. Each data point expresses the mean ± standard deviation (SD) from four replicates; **b)** Fluorescence intensity at 20 min time point of the EtBr retention curve in presence of compound **4.** Statistical significance was calculated using a one-way analysis of ANOVA, followed by multiple comparison test ($p > 0.05$).

a)

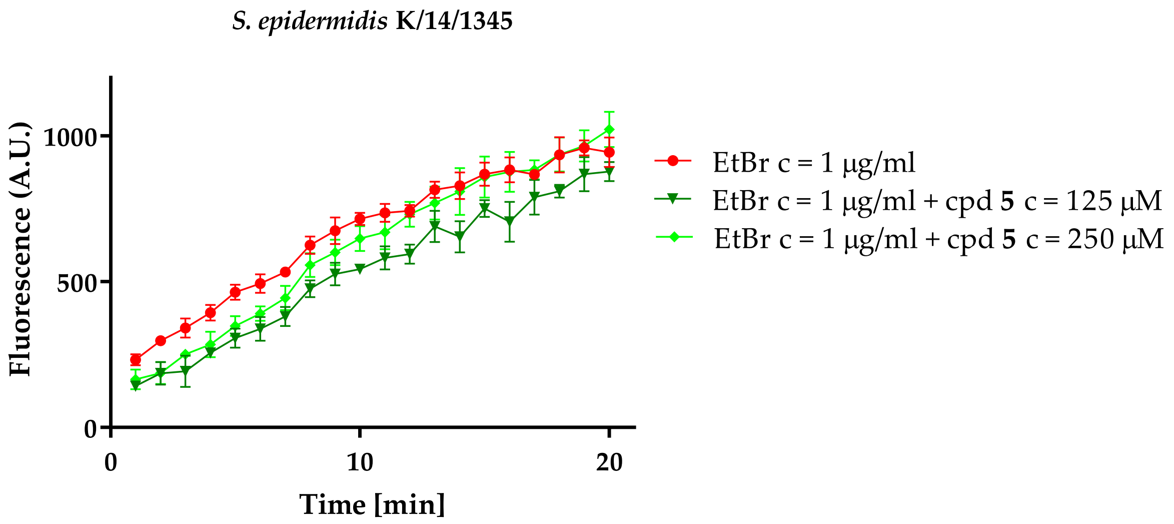

b)

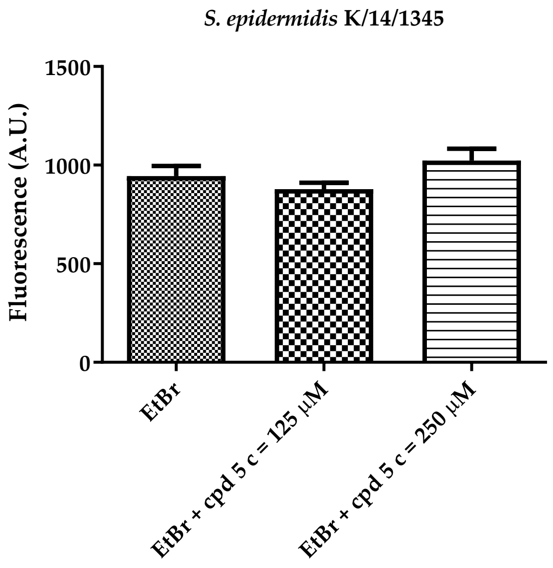

Figure 7S. a) Influence of compound **5** on accumulation of EtBr in *S. epidermidis* K/14/1345. Each data point expresses the mean ± standard deviation (SD) from four replicates; **b)** Fluorescence intensity at last (20 min) time point of the EtBr retention curve in presence of compound **5.** Statistical significance was calculated using a one-way analysis of ANOVA, followed by multiple comparison test ($p > 0.05$).

a)

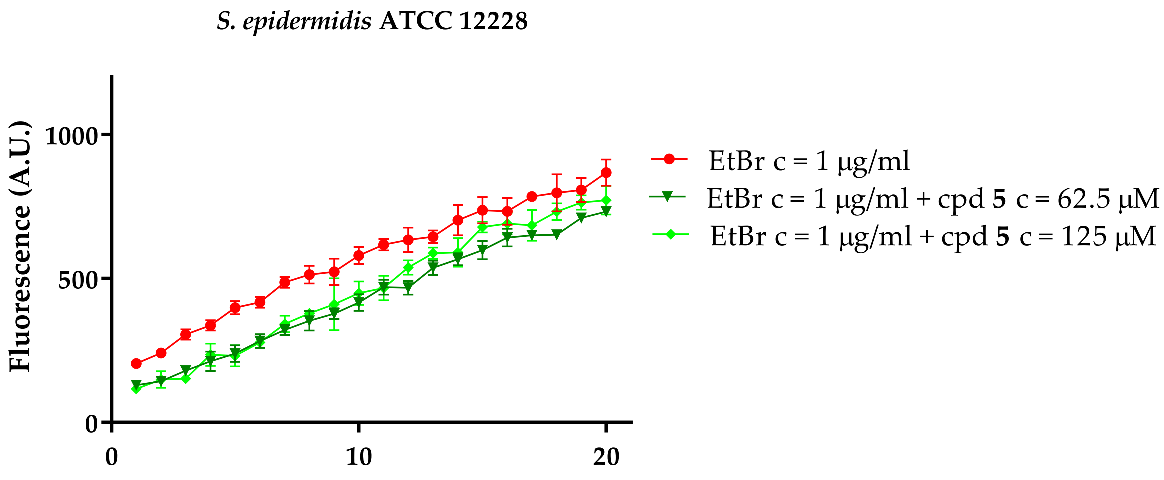

b)

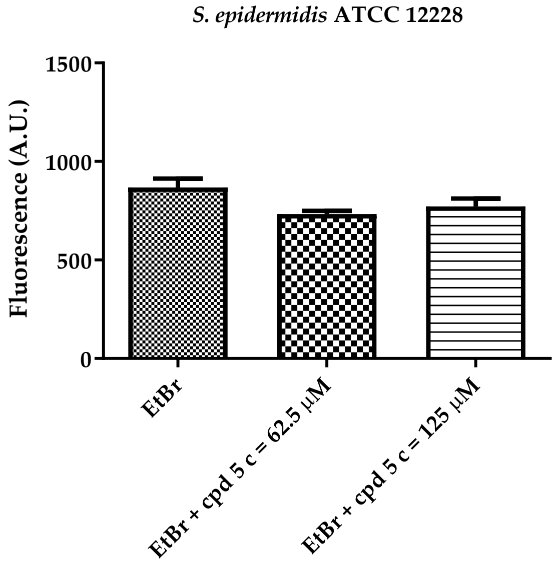

Figure 8S. a) Influence of compound **5** on accumulation of EtBr in *S. epidermidis* ATCC 12228. Each data point expresses the mean ± standard deviation (SD) from four replicates; **b)** Fluorescence intensity at 20 min time point of the EtBr retention curve in presence of compound **5.** Statistical significance was calculated using a one-way analysis of ANOVA, followed by multiple comparison test ($p > 0.05$).

a)

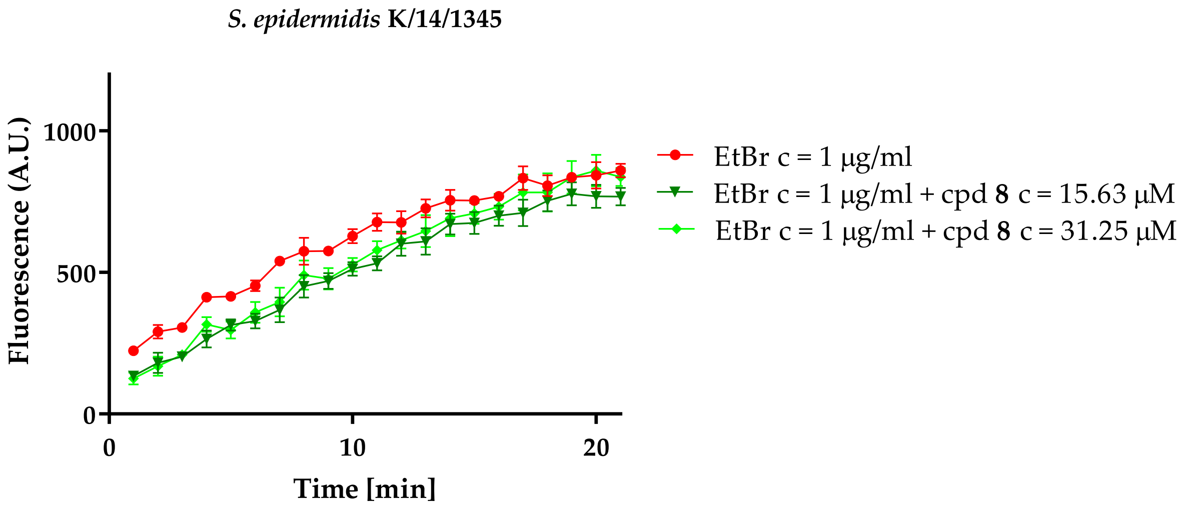

b)

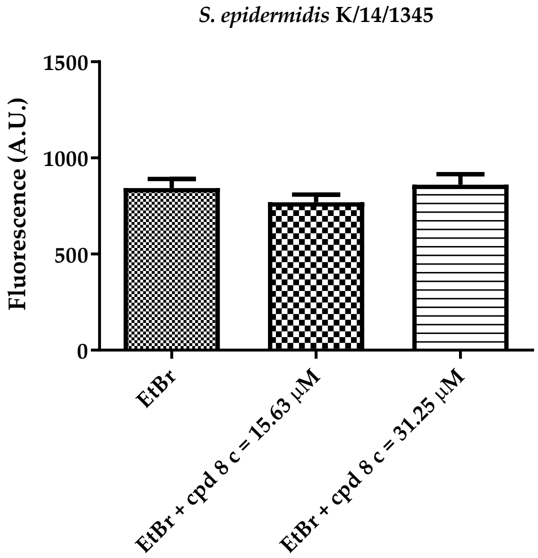

Figure 9S. a) Influence of compound compound **8** on accumulation of EtBr in *S. epidermidis* K/14/1345. Each data point expresses the mean ± standard deviation (SD) from four replicates; **b)** Fluorescence intensity at 20 min time point of the EtBr retention curve in presence of compound **8.** Statistical significance was calculated using a one-way analysis of ANOVA, followed by multiple comparison test ($p > 0.05$).

a)

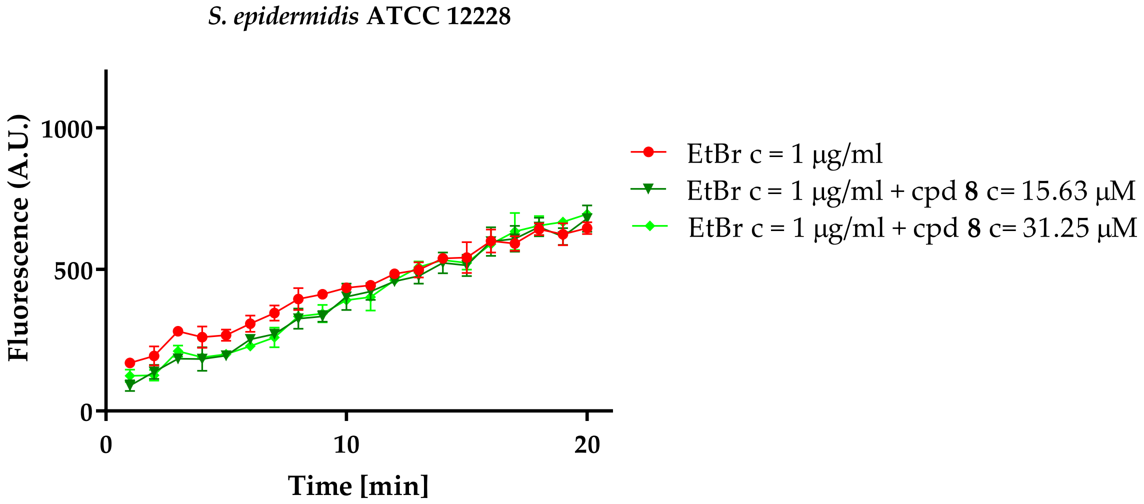

b)

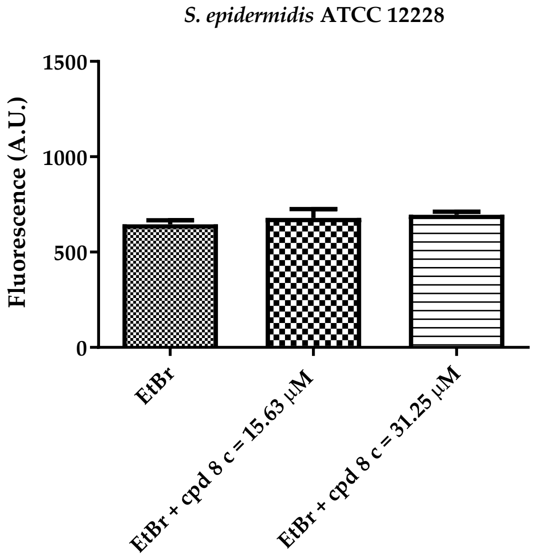

Figure 10S. a) Influence of compound **8** on accumulation of EtBr in *S. epidermidis* ATCC 12228. Each data point expresses the mean ± standard deviation (SD) from four replicates; **b)** Fluorescence intensity at 20 min time point of the EtBr retention curve in presence of compound **8.** Statistical significance was calculated using a one-way analysis of ANOVA, followed by multiple comparison test ($p > 0.05$).

a)

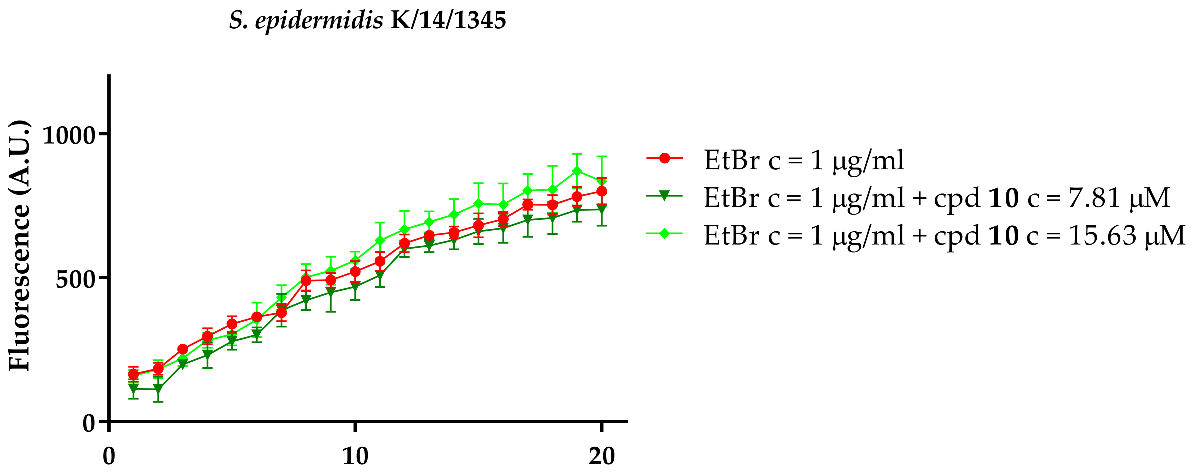

b)

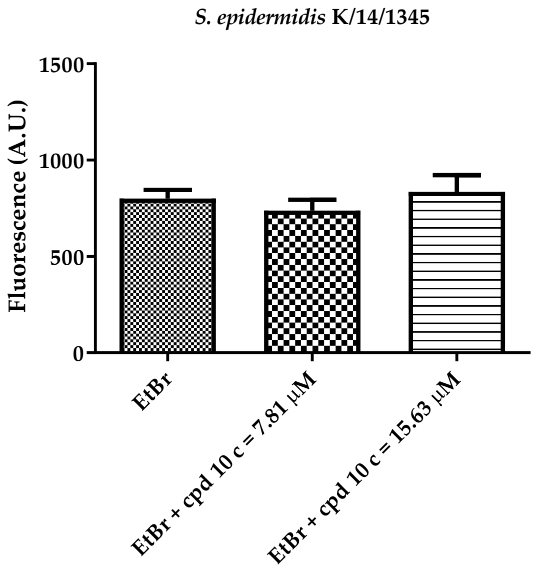

Figure 11S. a) Influence of compound **10** on accumulation of EtBr in *S. epidermidis* K/14/1345. Each data point expresses the mean ± standard deviation (SD) from four replicates; **b)** Fluorescence intensity at last (20 min) time point of the EtBr retention curve in presence of compound **10.** Statistical significance was calculated using a one-way analysis of ANOVA, followed by multiple comparison test ($p > 0.05$).

a)

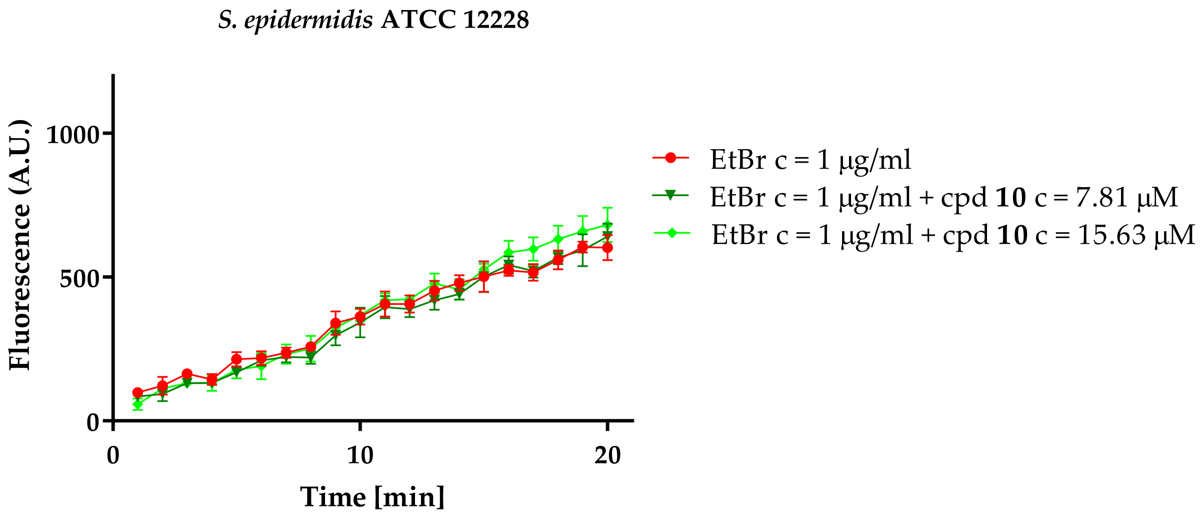

b)

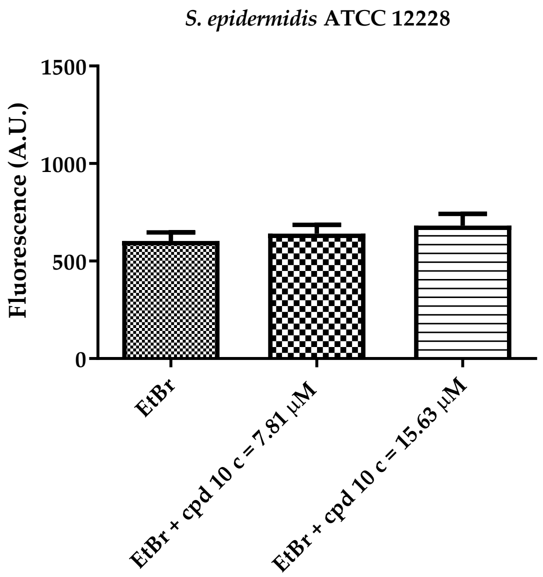

Figure 12S. a) Influence of compound **10** on accumulation of EtBr in *S. epidermidis* ATCC 12228. Each data point expresses the mean ± standard deviation (SD) from four replicates; **b)** Fluorescence intensity at 20 min time point of the EtBr retention curve in presence of compound **10.** Statistical significance was calculated using a one-way analysis of ANOVA, followed by multiple comparison test ($p > 0.05$).

a)

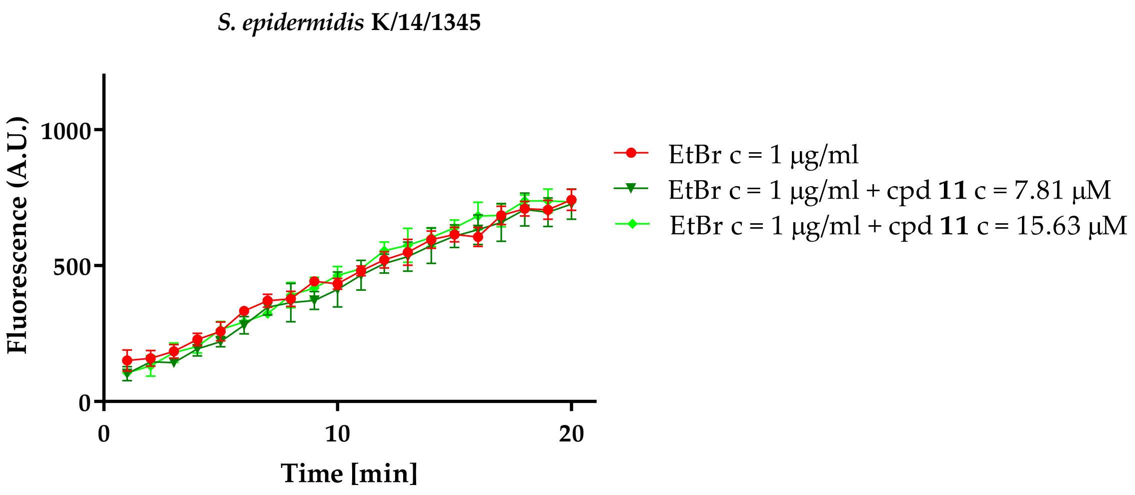

b)

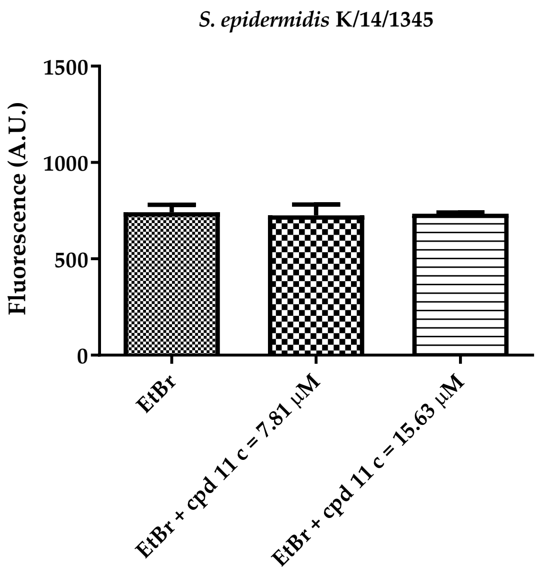

Figure 13S. a) Influence of compound **11** on accumulation of EtBr in *S. epidermidis* K/14/1345. Each data point expresses the mean ± standard deviation (SD) from four replicates; **b)** Fluorescence intensity at 20 min time point of the EtBr retention curve in presence of compound **11.** Statistical significance was calculated using a one-way analysis of ANOVA, followed by multiple comparison test ($p > 0.05$).

a)

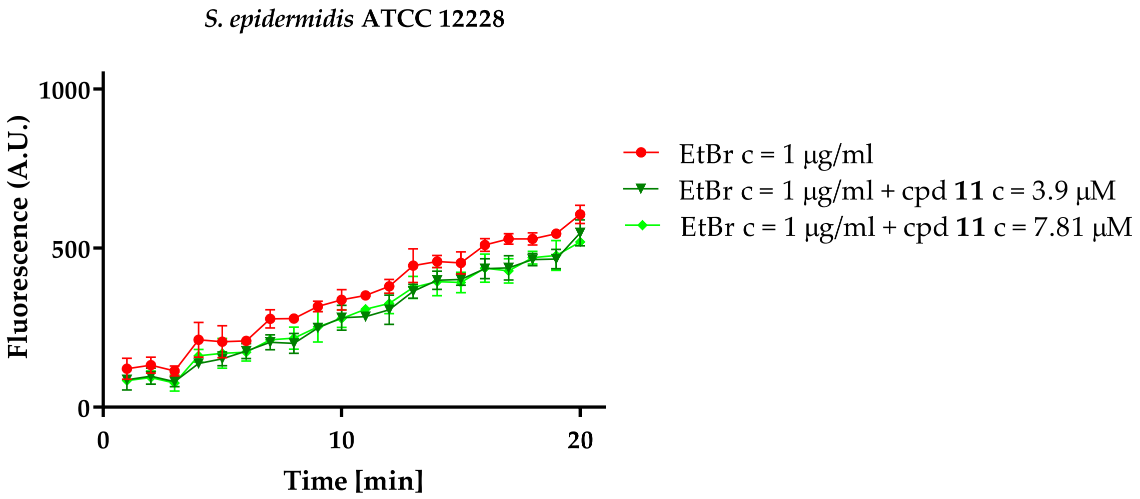

b)

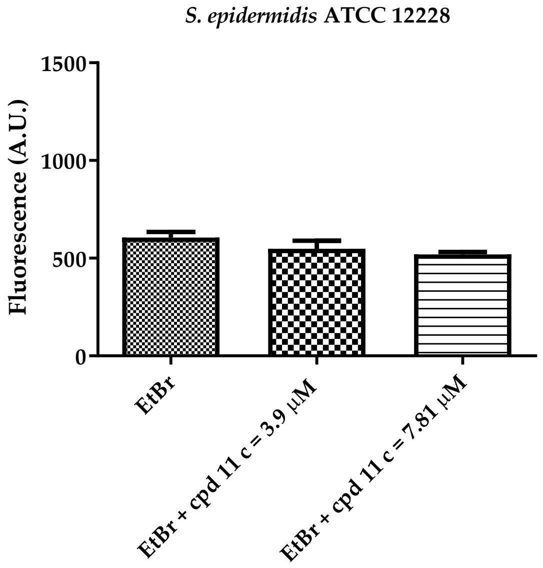

Figure 14S. a) Influence of compound **11** on accumulation of EtBr in *S. epidermidis* ATCC 12228. Each data point expresses the mean ± standard deviation (SD) from four replicates; **b)** Fluorescence intensity at 20 min time point of the EtBr retention curve in presence of compound **11.** Statistical significance was calculated using a one-way analysis of ANOVA, followed by multiple comparison test ($p > 0.05$).

a)

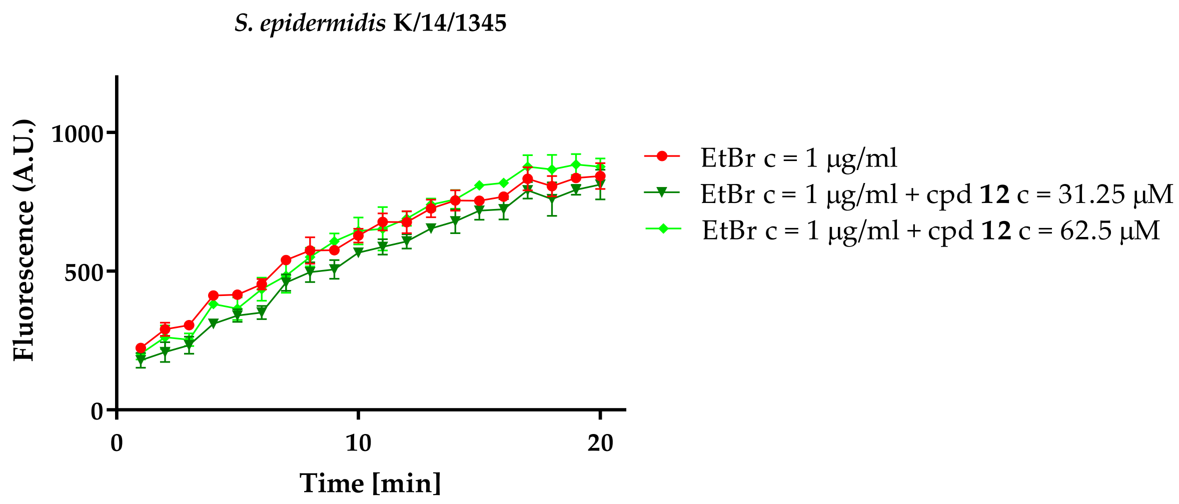

b)

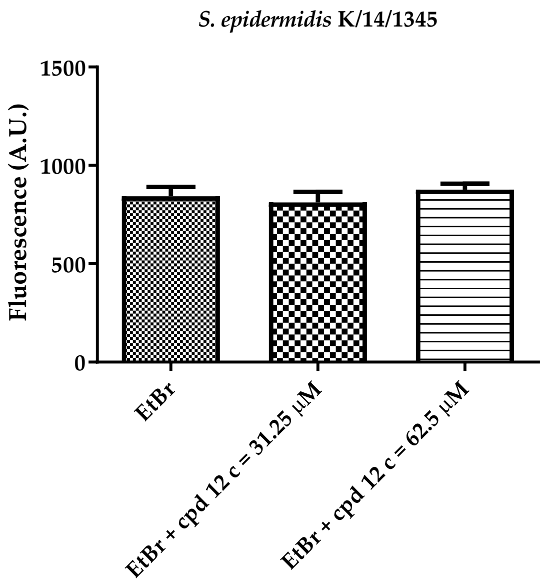

Figure 15S. a) Influence of compound **12** on accumulation of EtBr in *S. epidermidis* K/14/1345. Each data point expresses the mean ± standard deviation (SD) from four replicates; **b)** Fluorescence intensity at 20 min time point of the EtBr retention curve in presence of compound **12.** Statistical significance was calculated using a one-way analysis of ANOVA, followed by multiple comparison test ($p > 0.05$).

a)

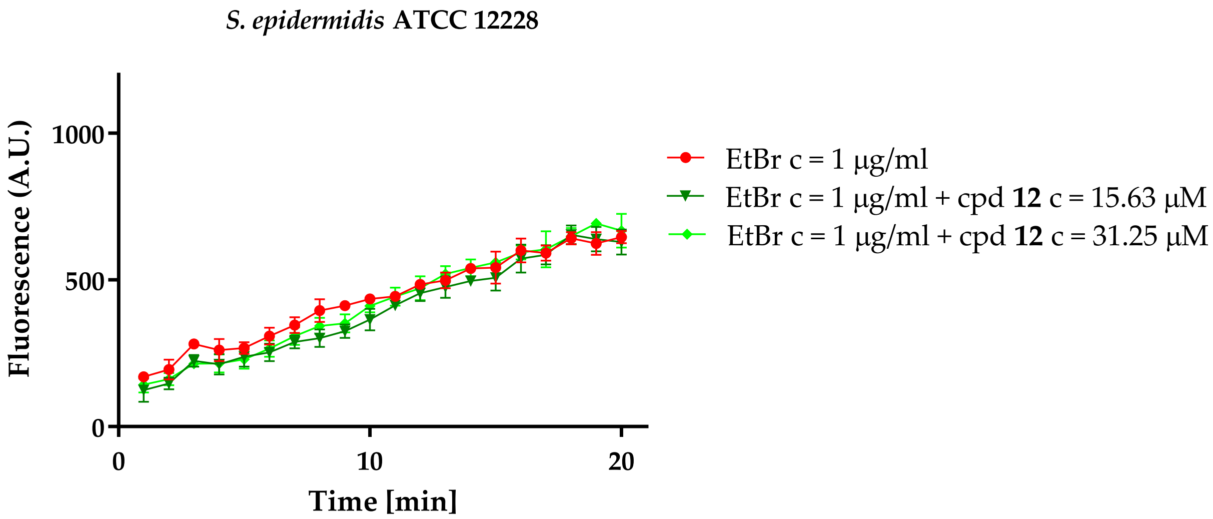

b)

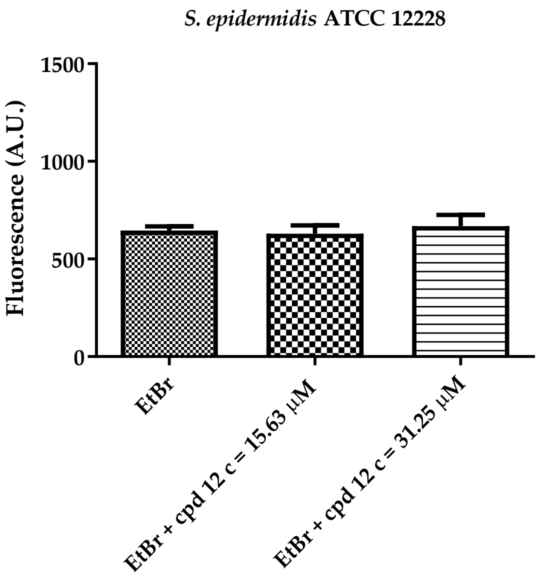

Figure 16S. a) Influence of compound **12** on accumulation of EtBr in *S. epidermidis* ATCC 12228. Each data point expresses the mean ± standard deviation (SD) from four replicates; **b)** Fluorescence intensity at 20 min time point of the EtBr retention curve in presence of compound **12.** Statistical significance was calculated using a one-way analysis of ANOVA, followed by multiple comparison test ($p > 0.05$).

a)

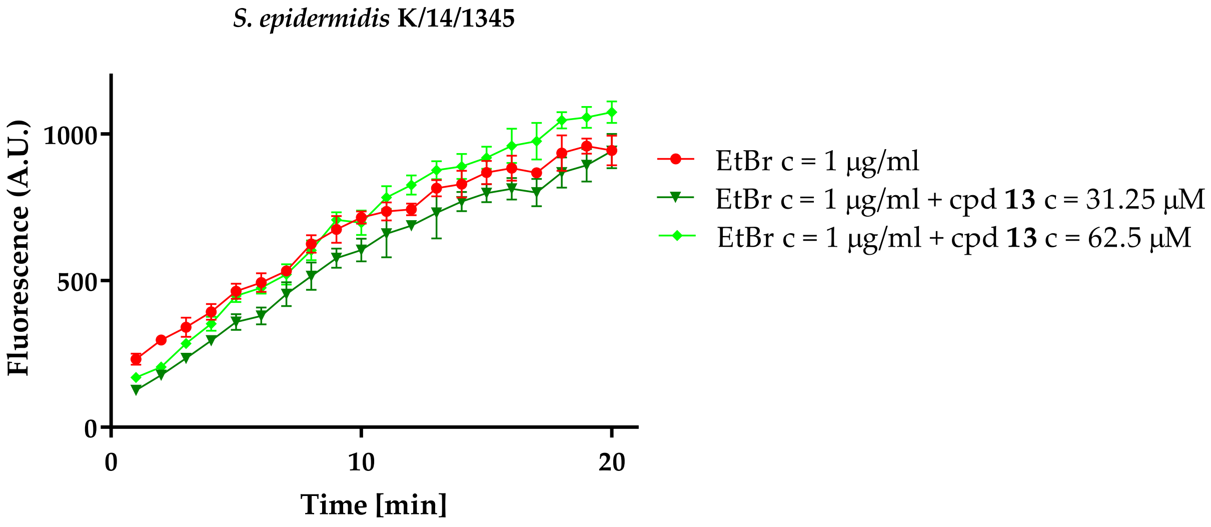

b)

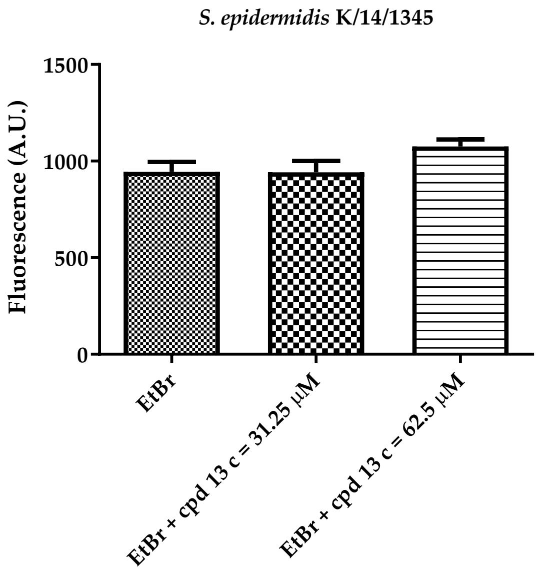

Figure 17S. a) Influence of compound **13** on accumulation of EtBr in *S. epidermidis* K/14/1345. Each data point expresses the mean ± standard deviation (SD) from four replicates; **b)** Fluorescence intensity at 20 min time point of the EtBr retention curve in presence of compound **13.** Statistical significance was calculated using a one-way analysis of ANOVA, followed by multiple comparison test ($p > 0.05$).

a)

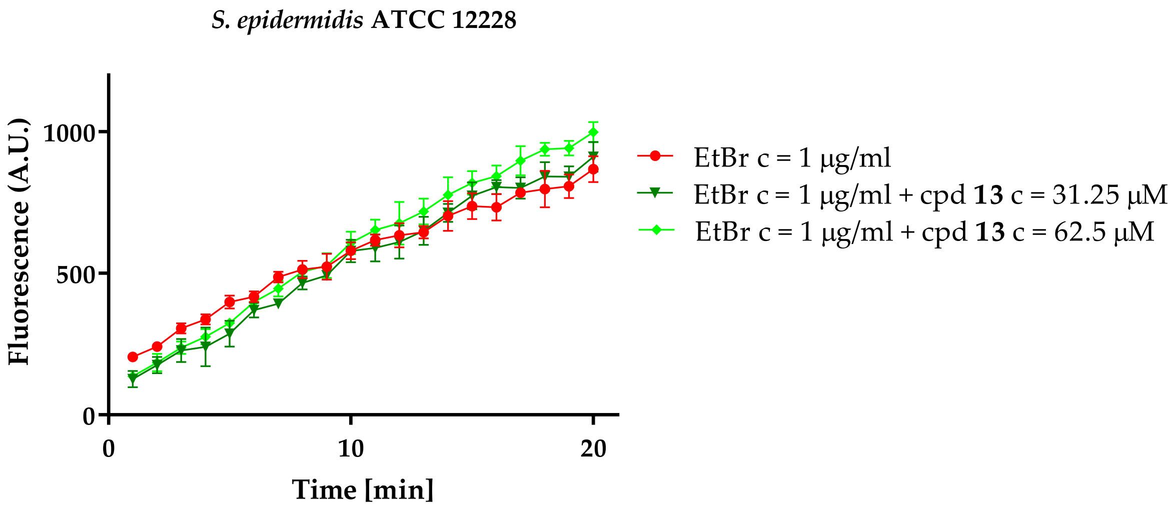

b)

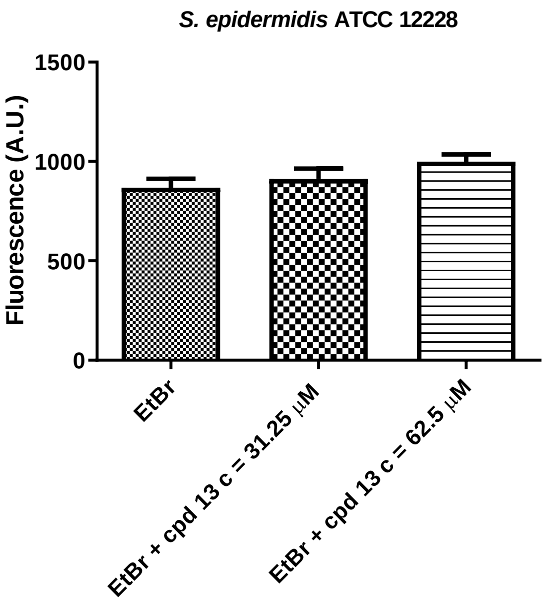

Figure 18S. a) Influence of compound **13** on accumulation of EtBr in *S. epidermidis* ATCC 12228. Each data point expresses the mean ± standard deviation (SD) from four replicates; **b)** Fluorescence intensity at 20 min time point of the EtBr retention curve in presence of compound **13.** Statistical significance was calculated using a one-way analysis of ANOVA, followed by multiple comparison test ($p > 0.05$).

a)

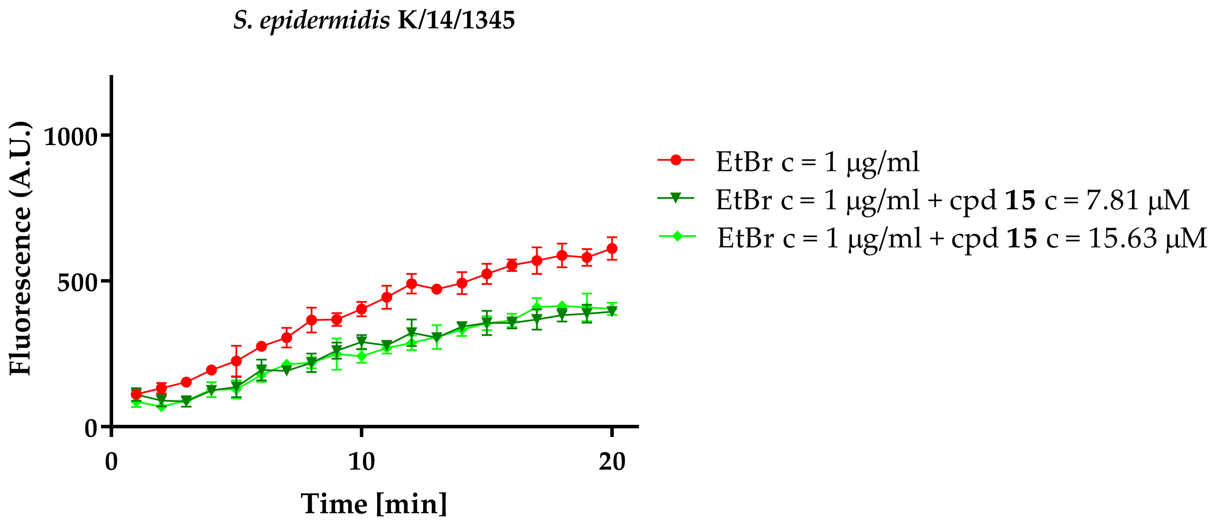

b)

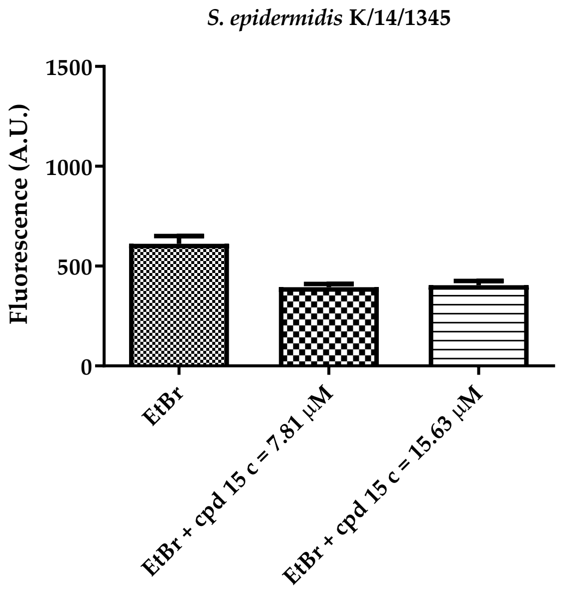

Figure 19S. a) Influence of compound **15** on accumulation of EtBr in *S. epidermidis* K/14/1345. Each data point expresses the mean ± standard deviation (SD) from four replicates; **b)** Fluorescence intensity at 20 min time point of the EtBr retention curve in presence of compound **15.** Statistical significance was calculated using a one-way analysis of ANOVA, followed by multiple comparison test ($p > 0.05$).

a)

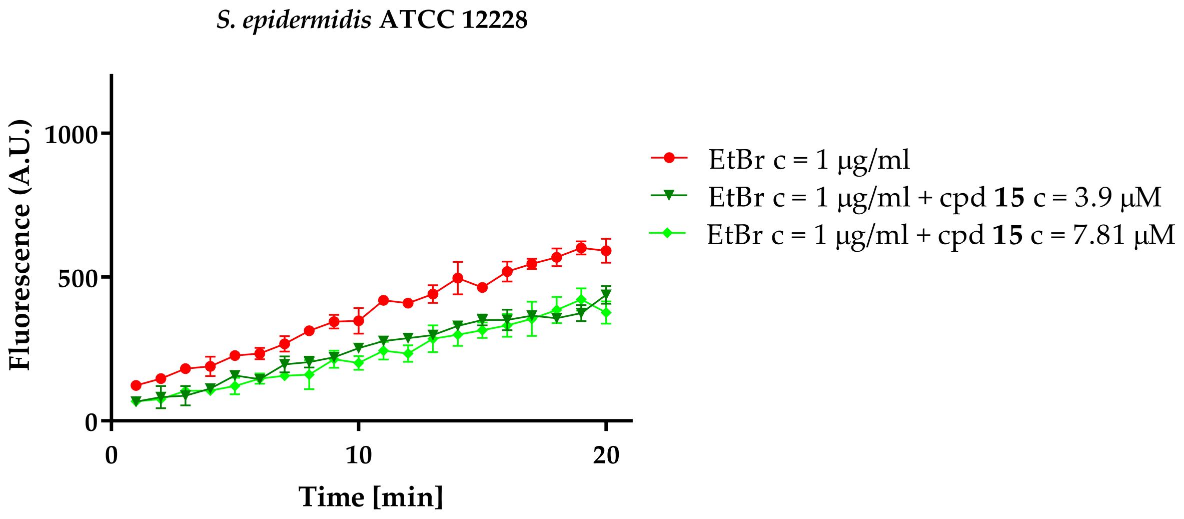

b)

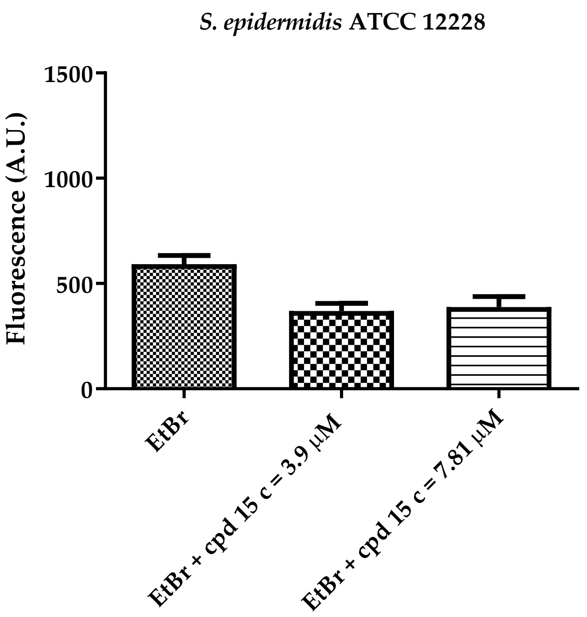

Figure 20S. a) Influence of compound **15** on accumulation of EtBr in *S. epidermidis* ATCC 12228. Each data point expresses the mean ± standard deviation (SD) from four replicates; **b)** Fluorescence intensity at 20 min time point of the EtBr retention curve in presence of compound **15** Statistical significance was calculated using a one-way analysis of ANOVA, followed by multiple comparison test ($p > 0.05$).
